# A Digitally Competent Health Workforce: Scoping Review of Educational Frameworks

**DOI:** 10.2196/22706

**Published:** 2020-11-05

**Authors:** Nuraini Nazeha, Deepali Pavagadhi, Bhone Myint Kyaw, Josip Car, Geronimo Jimenez, Lorainne Tudor Car

**Affiliations:** 1 Centre for Population Health Sciences Lee Kong Chian School of Medicine Nanyang Technological University Singapore Singapore; 2 Department of Primary Care and Public Health School of Public Health Imperial College London London United Kingdom; 3 Department of Public Health and Primary Care Leiden University Medical Center Netherlands Netherlands; 4 Family Medicine and Primary Care Lee Kong Chian School of Medicine Nanyang Technological University Singapore Singapore

**Keywords:** digital health, eHealth, health professions education, digital competency, competency, framework, review, medical education

## Abstract

**Background:**

Digital health technologies can be key to improving health outcomes, provided health care workers are adequately trained to use these technologies. There have been efforts to identify digital competencies for different health care worker groups; however, an overview of these efforts has yet to be consolidated and analyzed.

**Objective:**

The review aims to identify and study existing digital health competency frameworks for health care workers and provide recommendations for future digital health training initiatives and framework development.

**Methods:**

A literature search was performed to collate digital health competency frameworks published from 2000. A total of 6 databases including gray literature sources such as OpenGrey, ResearchGate, Google Scholar, Google, and websites of relevant associations were searched in November 2019. Screening and data extraction were performed in parallel by the reviewers. The included evidence is narratively described in terms of characteristics, evolution, and structural composition of frameworks. A thematic analysis was also performed to identify common themes across the included frameworks.

**Results:**

In total, 30 frameworks were included in this review, a majority of which aimed at nurses, originated from high-income countries, were published since 2016, and were developed via literature reviews, followed by expert consultations. The thematic analysis uncovered 28 digital health competency domains across the included frameworks. The most prevalent domains pertained to basic information technology literacy, health information management, digital communication, ethical, legal, or regulatory requirements, and data privacy and security. The Health Information Technology Competencies framework was found to be the most comprehensive framework, as it presented 21 out of the 28 identified domains, had the highest number of competencies, and targeted a wide variety of health care workers.

**Conclusions:**

Digital health training initiatives should focus on competencies relevant to a particular health care worker group, role, level of seniority, and setting. The findings from this review can inform and guide digital health training initiatives. The most prevalent competency domains identified represent essential interprofessional competencies to be incorporated into health care workers’ training. Digital health frameworks should be regularly updated with novel digital health technologies, be applicable to low- and middle-income countries, and include overlooked health care worker groups such as allied health professionals.

## Introduction

### Background

Over the last three decades, there has been considerable interest in the use of digital health to enhance the quality, efficiency, and safety of health care [1,2]. Digital health and eHealth are often used interchangeably and broadly defined as “the use of information and communications technology in support of health and health-related fields” [3]. An analysis of various digital health definitions revealed three distinct yet overlapping uses such as monitoring, tracking, and informing health (eg, mobile devices, mobile sensors and wearables, apps, social media); enabling health communication among various stakeholders (eg, telehealth, virtual consultations); and storing, managing, and utilizing health data (eg, electronic medical records, electronic medication systems) [4]. Digital health tools have the potential to provide health care workers with a holistic view of patients’ medical conditions through access to their health-related data and improved communication, regardless of distance and access [5]. Furthermore, the use of digital technology in health care could potentially reduce turnaround times, resource use, medication errors, and adverse drug events; increase the use of preventive care; and enable greater adherence to clinical guidelines [6-8].

Training and educating health care workers to be digitally competent is important for several reasons.

First, with the growing use of digital technology in health care, the roles and responsibilities of the health workforce are transforming in an unprecedented manner, intensifying the need for capacity building and continuous professional development. For example, a recent review commissioned by the United Kingdom Secretary of State for Health and Social Care (ie, the Topol Review) reported that within the next two decades, the majority of jobs in the National Health Service (NHS) will have a digital component [9]. Second, the importance and potential of remote care has been brought to light recently with the COVID-19 pandemic. Virtual consultation devices and electronic systems are indispensable tools used to diagnose and treat patients with potential COVID-19 infections as well as all other infections [10,11]. Third, even though the current and next generation of practitioners may be seen as “digital natives” [12], surveys of health care workers show that they would appreciate more training on digital technology [13,14]. Finally, improving digital literacy capabilities could lead to better adoption and implementation of digital services and technologies in health care settings [15]. Similarly, poor digital health literacy was found to be the most common barrier to the digital transformation of health care [16], and thus, the adoption of health technologies has been gradual in countries such as the United States [17], Europe [16], and Australia [18]. For the above-mentioned reasons, there is an increasing number of medical schools introducing digital health training into their curricula [12,19,20].

Such training programs should be guided by a clear framework of digital health competencies suited for different health care worker groups, settings, contexts, seniority, and role. Although there is an increasing number of individual digital health competency frameworks and reviews focusing on a specific health care worker role or setting [21-24], there is a need for consolidation, analysis, and a comprehensive overview of existing frameworks for all health care worker groups. This includes frameworks that are specific for and those that are relevant across different health professions, roles, or settings. Such an overview is important to inform increasingly interdisciplinary teams working in medicine and health care and corresponding future training initiatives, policy development, and research.

### Objectives

The objective of this review is to identify and analyze the available digital health competency frameworks, regardless of health profession, role, or setting. This scoping review takes into consideration the heterogeneity and complexity of this field, and we aim to identify (1) the intended applications of digital health competency frameworks; (2) the methodologies employed; (3) the targeted audience in terms of health professions and settings; and (4) the type of competencies included in the frameworks. By doing so, we aim to provide an in-depth analysis of the existing frameworks as well as identify potential gaps and propose recommendations for the development of future frameworks and digital health training initiatives.

## Methods

### Study Design

We followed the guidelines by the Joanna Briggs Institute [25] in performing a literature review and guidelines by Tricco et al [26] in creating a Preferred Reporting Items for Systematic Reviews and Meta-Analyses (PRISMA) flow chart. The protocol for this review was registered with the Open Science Framework [27]. In this review, we used the World Health Organization’s definition of digital health as “the combination of e-health and m-health as well as emerging areas, such as the use of advanced computing sciences in big data, genomics and artificial intelligence” [3].

### Eligibility Criteria

We included studies and reports focusing on the development and reporting of a digital health competency framework for health care workers. In this review, a competency framework is defined as a repository or a model that identifies, enlists, structures, and organizes competencies into meaningful categories and that has been developed via a systematic methodology or a relevant, established organization. This definition was developed *a priori* by referring to the existing definitions and descriptions, and looking at the common features of formerly identified digital health competency frameworks applicable to health care workers [28]. We included studies and reports on all health professions, including pre- and in-service, health care settings, and languages. Studies before January 2000 were excluded because digital health has evolved at a rapid pace, with substantial changes over the last two decades. The details of the inclusion and exclusion criteria are listed in Multimedia Appendix 1.

### Search Strategy

The search strategy was developed collaboratively and iteratively by the reviewers with support from an experienced medical librarian and was guided by the following: (1) the sensitivity of the search strategy to relevant articles identified from previous manual searches and (2) the total number of relevant results in the first few pages of results in Medical Literature Analysis and Retrieval System Online (MEDLINE) and EMBASE (Excerpta Medica dataBASE). The final MEDLINE search strategy (Multimedia Appendix 2) was translated to other databases. Subsequently, the following 6 databases were searched on November 8, 2019: MEDLINE, EMBASE, Cumulative Index to Nursing and Allied Health Literature (CINAHL), Education Resources Information Center (ERIC), PsycINFO, and the Cochrane Library. MEDLINE, EMBASE, ERIC, and PsycINFO were accessed via the Ovid platform, and CINAHL was accessed via EBSCOhost. We expected that there would be substantial unpublished work in this area, for which searches were performed using pertinent keywords (Multimedia Appendix 3) in OpenGrey, ResearchGate, and the first 10 pages of Google and Google Scholar. Websites of relevant professional associations (eg, International Medical Informatics Association [IMIA]), accreditation councils (eg, the US Accreditation Council for Graduate Medical Education [ACGME]), and key government websites (eg, Digital Health Canada, NHS Digital) were also searched (Multimedia Appendix 4).

### Screening and Data Extraction

The reviewers screened the search results and assessed the full-text studies for inclusion. For the title and abstract screening, Covidence tool [29], a web-based software platform, and, for full-text screening, EndNote X8 were used. Subsequently, a data extraction form was used to record information from the selected full-text studies using Microsoft Excel. The form was developed to be in line with the research objectives and was piloted by reviewers on 3 studies. The form was further amended (Multimedia Appendix 5), and relevant data were extracted by reviewers. Each round of screening and data extraction process was performed by a pair of reviewers independently, and results were compared thereafter. Disagreements between reviewers were resolved by discussion, and where required, a third reviewer was engaged as an arbiter.

### Data Synthesis

We analyzed the identified digital health competency frameworks in terms of their coverage of health professions, education level (eg, preservice or in-service), geographical applicability (ie, local or organizational, regional, national, international), health care settings (eg, acute care, emergency care, primary care), and other comparable features such as source and methodology. In frameworks that did not specify their methodology, an additional internet search was performed to retrieve information on the methods employed for framework development. Following this, we narratively synthesized and described framework characteristics, the evolution of frameworks over time, and the structural composition of competencies.

In addition, we performed a thematic analysis according to a list of steps proposed by Nowell et al [30] to understand the types of digital health competency categories presented in the frameworks. From the included frameworks, 2 reviewers independently classified competency categories into overarching domains. Studies that did not publish the full version of the framework were excluded from the thematic analysis, together with competency categories that were aimed at non–health care workers in a health care setting or irrelevant to digital competencies. Frameworks with ambiguous categories were excluded unless pertaining category descriptions or competencies were provided. Frameworks with competency themes or statements, with no distinct categories, were also included in the analysis by carefully allocating them to the identified domains. In some cases, where a single competency statement or theme encompassed several components of a competency, it was allocated to more than one relevant domain. After discussion and consensus, the reviewers derived a consolidated list of identified domains and their definitions and the prevalence of each identified domain across the included frameworks.

## Results

### Study Characteristics

**Figure 1:**
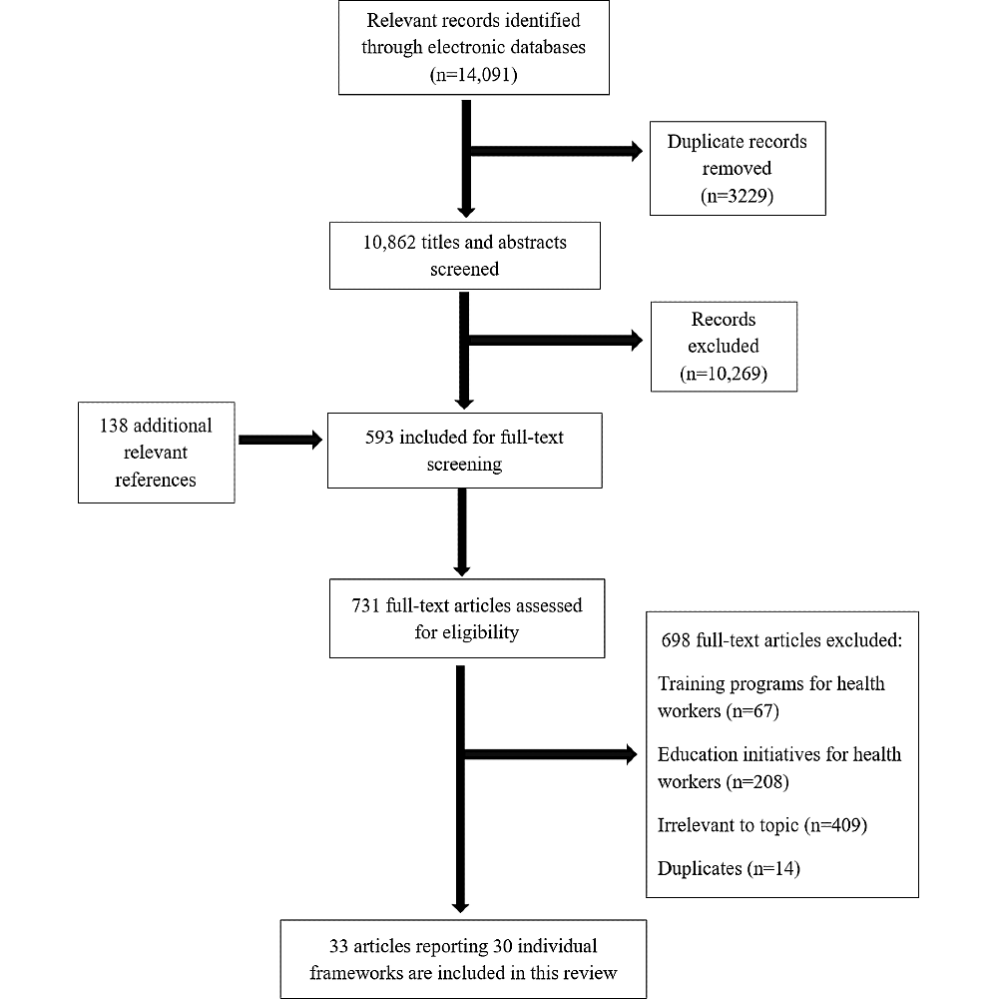
PRISMA (Preferred Reporting Items for Systematic Reviews and Meta-Analyses) flow chart.

The search generated 14,229 articles, of which 14,091 were from database searches and 138 from gray literature. After duplicates were removed and screening was completed, 33 articles were deemed eligible for inclusion. Of these, 27 articles presented individual frameworks. The remaining 6 articles presented preliminary findings followed by finalized versions of their frameworks (Staggers et al [31,32]; Egbert et al [33,34]; Hubner et al [35,36]), adding 3 more individual frameworks. As a result, a total of 30 frameworks are presented in this review (Figure 1), of which 16 were found through a gray literature search.

In terms of methodology, 14 frameworks employed literature review as an initial step, subsequently finalizing the frameworks through the use of expert consultations (n=5) [37-41], focus group discussions (n=3) [33,34,42,43], Delphi methodology (n=5) [31,32,44-47], or expert surveys [48]. In total, 11 other frameworks used one or a combination of methodologies (ie, Delphi, expert discussions, workshops, surveys) to reach consensus, largely by engaging various experts such as informaticists, health professionals, educators, and academics [21,22,36,49-56]. Of the remaining frameworks, 2 used only literature review to select relevant competencies [57,58], and 3 frameworks were built on the foundation of other published frameworks [59-61]. In addition, the frameworks were developed by a team of authors from a single university or institute (eg, University of Minnesota, School of Nursing) [44], by an international- or national-level organization (eg, IMIA, Australian Health Informatics Council [AHIEC]) [49,57], or by means of a collaborative effort to produce frameworks such as Technology Informatics Guiding Education Reform (TIGER) [35,36,52] and Health Information Technology Competencies (HITCOMP) [41].

In terms of geographical relevance, 15 frameworks were country-specific [21,33,37,38,40,42,45,47,48,50,55-59], 1 was specific to the European Union region [61], 5 were applicable globally [35,36,41,49,52,54], and the remaining did not specify (n=9). In terms of health care settings, 4 were developed for remote care delivery [39,51,53,60], 1 framework each for hospitals [48], acute care [41], and homecare [50], while the remaining frameworks either were applicable to all health care settings (n=5) [42,45,56,57,59] or did not specify (n=18). In terms of health professions, 14 frameworks targeted nurses, 4 targeted doctors, of which one also included dentists, and 1 framework each targeted psychiatrists, dietitians, and public health professionals; 9 frameworks targeted health care workers in general, of which 5 specified the inclusion of administrative, information technology (IT) support, and health informatics specialist roles [41,52,54,57,61], and 1 specified the inclusion of allied health professionals [39]. Among the nursing frameworks, 7 were meant for in-service nurses [21,31,32,42-44,46,48], 5 for preservice nursing students [33-37,53,58], and 2 for both [22,50]. Of the 4 medicine-focused frameworks, one focused on in-service doctors [40], another on preservice medical students [55], and 2 on both [59,60]. The framework characteristics and summary of the findings of the included studies are presented in Table 1 and Table 2, respectively. Additional details of the included studies are presented in Multimedia Appendix 6 [21,22,31-63].

**Table 1:** Characteristics of the 30 frameworks.

Characteristics		Values, n (%)
**Publication dates**		
	2000-2010	6 (20)
	2010-2019	24 (80)
**Source**		
	Database	14 (47)
	Gray literature	16 (53)
**Geographical setting**		
	Country	15 (50)
	Region	1 (3)
	International	5 (17)
	Not specified	9 (30)
**Health care setting**		
	Acute care	1 (3)
	Home care	1 (3)
	Hospitals	1 (3)
	Remote care	4 (13)
	All health care settings	5 (17)
	Not specified	18 (60)
**Health care profession**		
	Nursing	14 (47)
	Medicine	4 (13)
	Allied health	1 (3)
	Psychiatry	1 (3)
	Public health professionals	1 (3)
	Unspecified or applicable to multiple health professions	9 (30)
**Educational level**		
	In-service	13 (43)
	Preservice	9 (30)
	Both	8 (27)

**Table 2:** Summary of findings of included studies.

Study ID	Context (country; health care setting)	Intended audience (profession; educational level)	Methodology	Organization of framework
Academy of Medical Royal Colleges (2011) [59]	Scotland; all	Doctors and dentists; preservice and in-service	Framework aligned with other competency frameworks	418 competencies divided into 20 domains, and further subdivided into outcomes (ie, knowledge, skills, behavior)
AHIEC^a^ (2011) [57]	Australia; all	All HCPs^b^ including admin and IT^c^ support staff; preservice and in- service	Literature review drew on a wide range of major initiatives	45 competencies grouped into 3 categories and assigned a competency level ranging from 1 to 6
AFMC^d^ in Partnership with Canada Health Infoway (2014) [55]	Canada; not specified	Medical students; preservice	Framework was based on contributions from experts	25 competencies classified according to physicians’ roles and each competency is further divided into preclinical and clerkship milestones
Australian Nursing and Midwifery Federation (2015) [42]	Australia; all	Nurses and midwives (registered nurses, midwives, and enrolled nurses); in-service	Literature review Consensus via focus groups, on-line survey, and expert interviews	53 competencies grouped into 3 categories and 10 subcategories
Ayres (2012) [45]	United States; all	Registered dietitians, and dietetic technicians, registered and students; preservice and in-service	Literature review Delphi study	216 competencies grouped into 3 categories for each level of practice
Barakat (2013) [50]	The Netherlands; home care	Nurses; pre-service and in-service	A two-day collaborative workshop with academic experts	14 competencies organized into 5 themes
Brunner (2018) [47]	Australia; not specified	Health graduates; preservice	Literature review Focus group discussion Delphi study	40 competencies organized into 4 domains and further divided into knowledge and performance cues
Chang (2011) [21]	Taiwan; not specified	Nurses; in-service	3 web-based Delphi rounds conducted with experts	318 competencies grouped into 3 categories for each level of practice
Collins (2017) [46]	Not specified	Nurse leaders; in-service	Literature review Delphi study (3 rounds) Exploratory factor analysis for scale optimization and factor identification	74 competencies organized into 15 categories, and 15 of the most relevant competencies are ranked.
Crawford (2016) [51]	Not specified; remote care	Psychiatry residents; in-service	Expert panel and interviews with faculty and psychiatry residences	15 competencies sorted by professional roles, with examples
Curran (2003) [22]	Not specified	Nurse practitioners; preservice and in-service	Consensus with a team of nurse informaticists and nurse practitioner program directors	32 competencies grouped into 3 categories
Egbert (2016) [33]; Egbert (2019) [34]	Austria, Switzerland, Germany; not specified	Nurses; preservice	Literature review Expert survey Focus group discussion and consensus	24 competency areas identified, and 5 of the most relevant areas ranked for 5 nursing roles
HITCOMP^e^ (2019) [41]	International; acute care	All HCPs; preservice and in-service	Literature review Survey sent to international experts Gap analysis Expert consultation	1025 competencies organized into 33 competency areas, for 5 levels of practice across 5 domains
Hilty (2015) [60]	Not specified; remote care	Doctors, medical students; preservice and in-service	Competencies organized using the US ACGME framework, with input from the CanMEDS framework	Competencies listed for 8 main categories and subcategories, for each level of practice
Honey (2018) [58]	New Zealand; not specified	Registered nurses; preservice	Curriculum mapping Literature review	4 domains identified, and relevant subcategories and examples presented for each domain
Hubner (2016) [35]	International; not specified	Nurses; preservice	Survey sent to international experts A workshop was held to validate competencies	24 competency areas identified, and 6 of the most relevant areas ranked for 5 nursing roles
Hubner (2018) [36]	International; not specified	Nurses; preservice	Survey sent to international experts A workshop was held to validate competencies	24 competency areas identified, and sorted into 6 overarching domains 10 of the most relevant areas ranked for 5 nursing roles
Hubner (2019) [52]	International; not specified	All HCPs; preservice	Adapted Hubner (2016 and 2018)’s work [35,36] to include more HCP roles Survey was sent to international experts	33 competency areas are identified, and the 10 most relevant competencies are ranked for each HCP role
Hwang (2008) [48]	Taiwan; hospital	Clinical nurses; in-service	Literature review Expert survey	49 competencies grouped into 3 main categories and subcategories
Jidkov (2019) [40]	United Kingdom; not specified	Doctors; in-service	Literature review Curricular content analysis Expert consultation	20 competencies organized into 6 domains
Maheu (2018) [39]	Not specified; remote care	All HCPs including allied health professionals; Preservice and in-service	Literature review Expert consultation	7 domains identified, which are further broken down into 51 telebehavioral objectives, followed by 149 telebehavioral practices across 3 levels
Mantas (2010) [49]	International; not specified	All HCPs; in-service	Recommendations were discussed and refined by the IMIA^f^ task force.	34 competencies organized into 3 BMHI^g^ domains and each competency is determined if it is required by an IT user or a BMHI specialist according to 3 proficiency levels
Nagle (2014) [37]	Canada; not specified	Registered nurses; preservice	Literature review Consensus with experts through 3 rounds of feedback	19 competencies grouped into 3 domains.
NHS (2018) [56]	United Kingdom; all	All HCPs; in-service	Consultations with different stakeholders and workforce groups [62]	An overarching domain broken down into 5 domains, each with a set of description and competencies sorted according to 4 proficiency levels
Public Health Informatics Institute (2016) [38]	United States; not specified	Public health professionals; in-service	Literature review Expert consultation	8 categories identified, each with a set of competency statement and competencies
JASEHN (2018) [61]	Region; not specified	All HCPs including admin and IT support staff; in-service	Literature review Framework aligned with roles and competences as per the European eCompetence Framework Use of framework descriptions to determine skill level for each role [63]	Model 1: mission and main tasks described for 3 main profiles of health care workers (ie, health, nonhealth, and IT) Model 2: 52 competencies grouped into 6 domains, where each competency has a description, and a set of associated knowledge and skill, according to 5 proficiency levels
Staggers (2001) [31]	Not specified	Nurses; in-service	Literature review Consensus with experts	304 competencies grouped into 3 categories for each level of practice
Staggers (2002) [32]	Not specified	Nurses; in-service	Follow-up from Staggers (2001) [31] 3 Delphi rounds were conducted	281 competencies grouped into 3 categories for each level of practice
Thye (2018) [54]	International; not specified	All HCPs including admin and IT support staff; preservice	Mapped competency areas from Hubner (2018) [36] and HITCOMP [41] Survey was sent to HCPs	33 competency areas identified, and the 10 most relevant interprofessional areas are listed
Trangenstein (2009) [43]	Not specified	Nurse scholars; in-service	Literature review Discussion and consensus	7 competency domains sorted according to nursing level of practice
Van Houwelingen (2016) [53]	Not specified; remote care	Nurses; preservice	Survey with competencies was sent to participants Delphi study (4 rounds)	52 competencies organized into 3 categories, where skills are further subdivided into 5 domains
Westra and Delaney (2008) [44]	Not specified	Nurse leaders; in-service	Literature review Delphi study	92 competency areas are grouped into 3 categories

^a^AHIEC: Australian Health Informatics Education Council.

^b^HCP: health care professional.

^c^IT: information technology.

^d^AFMC: Association of Faculties of Medicine of Canada.

^e^HITCOMP: Health Information Technology Competencies.

^f^IMIA: International Medical Informatics Association.

^g^BMHI: biomedical and health informatics.

### Evolution of Frameworks

**Figure 2:**
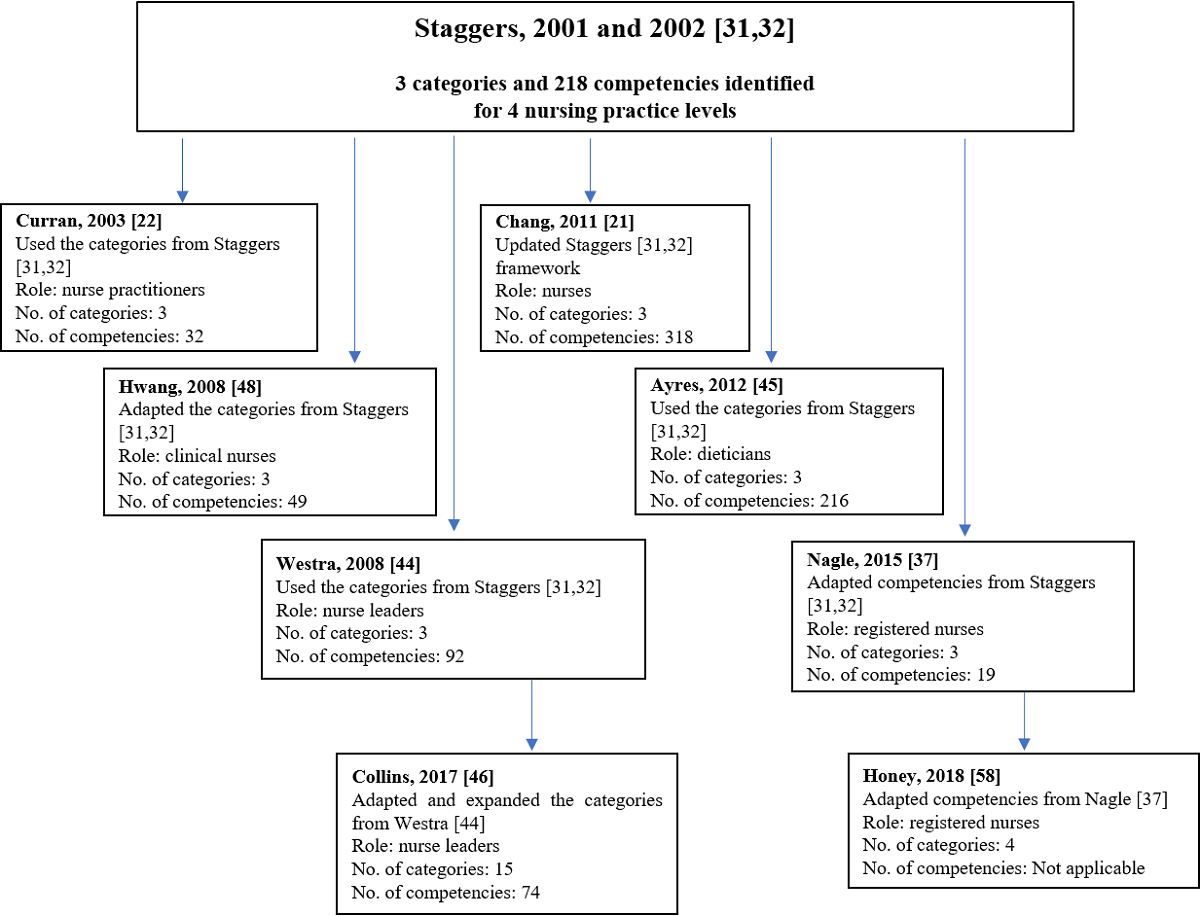
Digital health competency frameworks adapted from Staggers framework.

**Figure 3:**
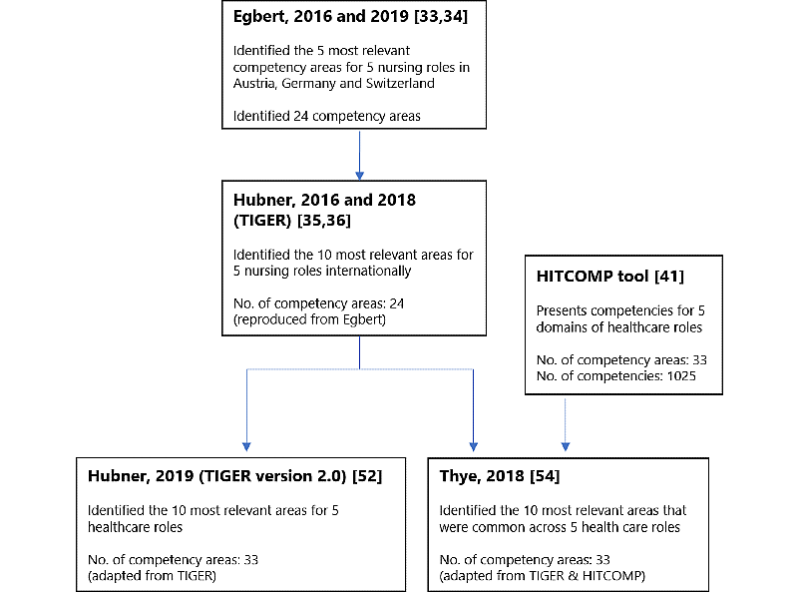
Development of recent digital health competency frameworks. HITCOMP: Health Information Technology Competencies; TIGER: Technology Informatics Guiding Education Reform.

The digital health competency frameworks have drawn upon each other and have incrementally advanced the recommendations made in this area, as presented in Figures 2 and 3. The initial work of Staggers et al [31,32], which targeted nurses at 4 levels of practice, has been reproduced and adapted to suit different nursing roles (eg, nurse leaders, nurse practitioners) [22,44], health professions (eg, dietitians) [45], and even geographical settings (eg, Taiwan, Canada; Figure 2) [37,48]. In another instance, to propose competencies for nurse leaders, Collins et al [46] expanded and reorganized competency categories from Westra and Delaney [44], which initially drew inspiration from the framework by Staggers et al [31,32]. The framework by Staggers et al [31,32] was updated 10 years later by Chang et al [21] with 42 new competencies.

Another commonly referenced framework is that by Egbert et al (Figure 3) [33,34]. This framework identified 24 core competency areas and conducted a survey with experts to rank the most relevant competency areas for nurses in Austria, Switzerland, and Germany. The same survey with 24 core competency areas was then sent to multiple countries to put forth international recommendations for nursing informatics, widely known as the TIGER framework [35,36]. The TIGER framework grouped the core competency areas into 6 overarching domains (ie, data, information and knowledge, information exchange and information sharing, ethics and legal issues, systems life cycle management, management in informatics, biostatistics, and medical technology) and ranked the most relevant competencies for nursing internationally. Subsequently, the TIGER framework prompted its 2.0 version to include a wider spectrum of health care workers [52], and inspired the work by Thye et al [54] where interprofessional competencies were identified.

One framework that was accessible through an open-source internet-based database is the HITCOMP Tool and Repository [41]. HITCOMP was produced by the eHealth Workforce Development Workgroup as part of the EU*US eHealth Work Project. Its overall goal was to map, quantify, and project the needs of a digitally competent workforce [64]. This tool covers the digital competencies required in acute care settings for 5 broad roles of health care workers, similar to the TIGER framework version 2.0 (ie, direct patient care; administration; engineering or information, communication, and technology; informatics; and research or biomedicine) [52]. In comparison with the other included frameworks, the HITCOMP framework has the highest number of competencies at 1025.

Other frameworks that inspired the development of subsequent frameworks include the IMIA [49], which was adapted by the AHIEC [57] to create national informatics standards for Australian health professionals, health informaticians, and specialists; the frameworks by Barakat et al [50] and the Academy of Medical Royal Colleges [59], which were adapted by Van Houwelingen et al [53] to develop a telehealth framework aimed at nurses; and the telepsychiatry framework by Hilty et al [60], which laid the foundation for telebehavioral health competencies by Maheu et al [39].

### Digital Health Competencies and Categories

Across the included digital health competency frameworks, the number of competency categories, subcategories, and competencies ranged from 3 to 33, 10 to 39, and 15 to 1025, respectively. The wide range of reported competencies reflects the scale and specificity of the frameworks. For example, the framework with 15 competencies was focused on telepsychiatry training for psychiatry residents [51], while a framework with 318 competencies was intended for nurses at 4 different levels of practice [21]. Frameworks with multiple categories or subcategories had a larger number of competencies. For example, the eHealth competency framework by the Academy of Medical Royal Colleges presented 20 categories and included 418 competencies [59].

The primary objective of the frameworks was to guide the development of digital health curricula or training initiatives. Thus, 20 frameworks listed specific competencies (eg, knows how to use medical information systems for retrieval of patient data) [48], 6 frameworks cited case studies or provided examples for integration of competencies into curricula or training programs [33-36,45,51,52,58], and 3 frameworks ranked the most relevant competency areas, while the remaining 2 frameworks proposed only broad competency domains. Furthermore, in 12 frameworks, proficiency level in a digital health area or competency was presented according to the hierarchy of a profession or according to the depth of a skill that can be acquired by a health care worker role. For example, Chang et. al [21] presented competencies according to nursing staff seniority level (ie, a beginning nurse, an experienced nurse, an informatics specialist, an informatics innovator). Conversely, AHIEC assigned each competency a level of 1 to 6 (ie, 1: Remembering; 2: Understanding; 3: Applying; 4: Analyzing; 5: Evaluating; 6: Creating), according to the revised Taxonomy of Learning Domain objectives by Bloom, to indicate the depth of a skill that can be acquired by a health care worker role [57].

In addition, the structure of the included digital health competency frameworks varied. Competencies were organized either based on broad informatics categories (ie, computer skills, informatics knowledge, informatics skills) [21,22,31,32,42,44,45,48,53] or a combination of informatics and noninformatics categories (ie, information systems concepts, management concepts, ethical or legal concepts) [38,41,46,59-61] or based on health care worker roles (ie, communicator, collaborator, professional) [33-36,51,52,54,55]. Alternatively, competencies were sorted according to learning outcomes or statements [37,39,40,43,47,49,50,56-58]. For example, the national guideline for Canadian registered nurses sorted competencies according to 3 overarching statements: (1) uses relevant information and knowledge to support the delivery of evidence-informed patient or client care; (2) uses IT in accordance with professional and regulatory standards and workplace policies; and (3) uses IT in the delivery of patient or client care [37].

Through thematic analysis, we were able to classify the majority of the competency categories presented in the frameworks into 28 domains. These domains are defined in Textbox 1, details of the analysis are provided in Multimedia Appendix 7 [45], and results of the analysis is presented in Multimedia Appendix 8. Competencies relating to the following domains were found to be prevalent in at least half of the included frameworks: informatics concepts and processes (22/30, 73%); health information and records management (19/30, 63%); communication (19/30, 63%); ethics, legal, or regulations (18/30, 60%); privacy and security (17/30, 57%); technical knowledge and support (15/30, 50%); and clinical care delivery (15/30, 50%). Conversely, competencies relating to medicines management (2/30, 7%) [41,59]; attitudes toward IT (4/30, 13%) [48,51,58,60]; IT advocacy (5/30, 17%) [21,22,51,55,60]; and public health (5/30, 17%) [41,49,52,54,55] were found to be uncommon in digital health competency frameworks. Of the 28 identified domains, 20 (71%) were present in at least one-third of the frameworks (Multimedia Appendix 8).

**Digital health competency domains identified from the included frameworks.**

Administration and general management

- Use of administrative information technology (IT) applications to perform tasks and procedures, such as planning and delivery of services, business workflows, and human resource management

Analysis

- Use of IT systems to perform analysis of data, including data visualization, evaluation, and reporting

Attitudes toward IT

- Attitudes and cultural awareness toward the use of IT in patient care

Clinical care delivery

- Utilization of IT for the support of clinical care and practice, including use of assistive technologies and electronic test requesting

Communication

- Use of digital communications (eg, social media, email, etc) to enhance interpersonal skills and to aid in care delivery and decision-making process

Decision support

- Use of IT for clinical practice decision support

Documentation

- Use of IT for appropriate documentation tasks and processes, including knowledge of coding and terminologies

Education and training

- Use of IT in education and training, including e-Learning, mobile learning, and simulation

Ethics, legal, or regulations

- Knowledge of ethical, regulatory, compliance, and legal requirements relating to health IT

Financial management

- Knowledge of financial and account management relating to IT applications, including billing and fiscal aspects

Health information and records management

- Ability to access, collect, store, share, and manage digital health information; use of eHealth records; information and knowledge management

Health care quality and safety

- Ensuring or improving the quality and safety of health care services with the use of IT

Imaging

- Knowledge of biomedical imaging digital technologies

Informatics concepts and processes

- Knowledge and skills in computer basics, information systems, and general health IT use

Integration and interoperability

- Knowledge of integrated health IT applications, health information exchange, and interoperability standards, including coordination and collaboration aspects

IT advocacy

- Play an active role in promoting the use of IT in health care environments

Leadership and executive management

- Providing or enhancing executive leadership skills relating to the use of IT, including setting direction, strategic management, change management, stakeholder management, and governance

Medicines management

- Management of digital medication records and use of order entry

Patient access and engagement

- Promoting use of IT applications among patients and supporting or empowering patients for self-management, including patient access to patient health records

Privacy and security

- Ensuring that digital data and health information are protected and kept confidential by following privacy and security procedures

Project management

- Knowledge of project management software and associated terminologies

Public health

- Use of IT to inform public health strategies

Remote care

- Provision of care at a distance, including telehealth care, eHealth, mobile health, and related fields

Research

- Appropriate use of IT for research support and innovations

Risk management

- Managing IT-related risks

Systems implementation

- Knowledge and skills about IT systems development, management, and implementation

Technical knowledge and support

- Knowledge of technical aspects of IT systems, including software applications, testing, applied computer science, and IT maintenance and support capabilities

## Discussion

### Principal Findings

Of the 30 digital health competency frameworks, 14 solely targeted nursing staff. The frameworks predominantly originated from high-income countries and were developed based on literature reviews, followed by expert discussions or a Delphi approach. More than half of the included frameworks, especially those providing national-level recommendations, were from gray literature sources. Most frameworks were published between 2016 and 2019, highlighting the growing interest in digital health competencies in recent years.

The purpose of the retrieved digital health competency frameworks and the intended audience was clearly stated in most frameworks. The earliest frameworks and almost half of all the included frameworks were meant for nurses. This could be due to the significantly larger proportion of nurses in the health workforce [65]. Nurses play a crucial role in supporting health care environments by being a constant point of contact between patients and doctors; thus, there are various aspects of digital capabilities that are required of them (eg, using staff scheduling systems, extracting data from clinical systems, navigating decision support systems) [22]. These nursing-focused frameworks have inspired subsequent works for other health care workers. For example, several competency areas subsumed under broad areas for nurses in the TIGER framework [36] were marked as standalone competency areas (ie, communication, legal issues, interoperability and integration, and life cycle management) for a wider spectrum of health care workers in version 2.0 of the TIGER framework [52]. Notably, only one framework was found for allied health professionals (ie, dietitians) [45], which highlights the perceived lack of interest in educating and training this group of workers in digital health. However, the Health Informatics Society of Australia, now known as the Australasian Institute of Digital Health, highlighted the need to focus on allied health care workers as their involvement is becoming increasingly important in decision making to improve patient care and health outcomes [66]. Furthermore, although there were frameworks for doctors and medical students collectively, only one framework was intended solely for undergraduate medical education. The framework was a national guideline for medical students in Canada briefly describing 25 eHealth competencies [55]. Medical practice relies heavily on communication, which is now achieved through various digital means; thus, the skills to utilize a range of digital technologies should be comprehensive and included in medical education [67]. Moreover, with the COVID-19 pandemic, doctors are required to handle patient consultations digitally [11]. A 2018 survey conducted by European Medical Students’ Association revealed that a majority of medical students rated their eHealth skills to be “poor” or “very poor” and desired for adequate digital health literacy [14].

Most of the included frameworks are useful for application in education or practice, mainly owing to the specificity of competencies, the organization of competencies according to proficiency levels or health care worker roles, and the illustration of case studies and examples to be applicable to various settings. On the other hand, frameworks by Trangenstein et al [43] and Jidkov et al [40] presented only broad competency themes, as it was believed that exhaustive lists of competencies could lead to poor adoption [40]. As the included digital health competency frameworks were heterogeneous in purpose, audience, and setting, it is challenging to determine a single framework as exemplary. Nevertheless, HITCOMP, which was developed via an iterative methodology, was found to be the most comprehensive framework, covering 21 out of the 28 identified competency domains, listing 1025 competencies, and targeting a wide audience of health care workers and medical specialties [41].

The key thrust of work in this area involves competencies related to informatics, followed by eHealth, telehealth or telebehavioral or telepsychiatry, digital capability, and health IT competencies. This distinction may be superficial, given that the definitions and terminologies seem to overlap across frameworks and the nomenclatural differences do not necessarily convey differences in competencies. For example, the interprofessional eHealth framework developed by Thye et al [54] utilized a range of informatics frameworks [33,41,49].

Although frameworks often drew upon each other, there were considerable variations among the identified competencies. The Egbert et al [33,34] framework identified the 5 most relevant competency areas (out of 24) for 5 nursing roles in Austria, Germany, and Switzerland. The TIGER framework [35,36] used the same competency areas and methodology as Egbert et al [33,34] and additionally reached out to experts worldwide for their inputs. The resulting relevant competency areas for a nurse in an IT management role, for example, varied considerably between both frameworks. For this role, competency areas, *risk management* and *project management*, ranked the top 5 internationally in the TIGER framework [35,36]; however, it was only relevant in 1 out of 3 countries in the Egbert et al [34] framework. In addition, eHealth, telematics, and telehealth, which were ranked as top 5 by Egbert et al [34] for 2 out of 3 countries (ie, Germany and Austria), did not make it to the top 10 list in the TIGER framework [35,36]. This suggests that the IT management role for nurses could be defined differently depending on each setting. Hence, a clear definition of the role is important to match the appropriate competency skills to a health care worker role.

In our thematic analysis of the competencies included in the retrieved frameworks, we identified 28 competency domains. The most prevalent domain relates to competencies aimed at providing knowledge on informatics concepts and processes. Examples of these include basic computer knowledge, information systems concepts, and principles of informatics, which are fundamental skills to health care workers intending to maximize the use of digital technologies. The other common domains included the ability of health care workers to manage data from health information systems and records and to be well-versed in digital communications. Most of the health-related data today exist in digital form; therefore, it is imperative for health care workers to be able to understand the purpose, basic structures, use, and storage of electronic health records (EHRs). In addition, as digital health entails new forms of communication (eg, virtual consultations, email, chatbots), it is imperative for health care workers to be able to relate information accurately yet efficiently, timely, and delicately to patients, colleagues, and other collaborating stakeholders [68]. Furthermore, the rise in the adoption and utilization of digital technologies has spurred new issues relating to the use of IT [69], which corresponds to the next two common domains of competencies (ie, ethics, legal, or regulations, and privacy and security). These domains stress the importance of health care workers’ adherence to legal and regulatory requirements and to keep up to date with privacy and security policies pertaining to the appropriate use of digital technologies.

Some categories that were found in more recent frameworks, such as attitudes toward the use of IT, medication and prescription, IT advocacy, and public health, reflect the emerging trends in digital capabilities required by health care workers. For example, with the widespread adoption of EHRs and e-prescribing being a key functionality, it is imperative for physicians to be able to perform prescribing tasks efficiently and adapt to new features as systems continually evolve [70]. In addition, as digital technologies have an increasing role in the management of health of communities and populations, frameworks have also started to incorporate competencies related to public health [71]. Similarly, the acceptance of IT and its incorporation into everyday practice hinges on health care workers advocating for their use and being mindful of contextual factors and beliefs that would enable their use in different settings, such as high-income and low- and middle-income countries (LMICs). Other distinct domains such as leadership, administrative, managerial, and financial bring to attention that a digitally competent workforce should also be able to utilize technologies to oversee organizational-level aspects. It was also noted that competencies related to artificial intelligence, robotics, and social media, which are very relevant in current times, were not explicitly mentioned in the included frameworks [5,9,72]. One possible explanation could be that some of these are subsumed under broader categories, for example, competencies regarding the use of social media could be part of the *communication* category, or that these digital areas have yet to be covered in digital health competency frameworks.

From this synthesis of digital health competency frameworks, we would like to propose recommendations for the development of future frameworks (Textbox 2). First, an iterative methodology that includes literature review and consultations with local and international experts is ideal for a comprehensive framework. Second, it is encouraged for upcoming frameworks to explore competency areas that appeared in more recent frameworks, to cover upcoming digital health areas (ie, health apps, artificial intelligence, autonomous decision-support systems), and to be open to future revisions to be up to date with technological developments. For example, HITCOMP is projected to continue mapping and aligning competencies with the curriculum and other major initiatives [64]. Finally, the lack of a comprehensive and international framework applicable to allied health professionals and LMICs warrants the development of frameworks that include these populations and settings. For example, only the TIGER framework includes case studies of LMICs, such as China, India, and Nigeria [73].

**Recommendations for the development of digital health competency frameworks.**

Methodology:

- Literature review, followed by consultations or Delphi study with local experts, followed by engagement with international experts

Content:

- Explore new competency areas that appeared in recent frameworks (ie, attitudes and advocacy toward the use of information technology (IT), medication management, public health)
- Update competencies based on technological innovations and adoption and emerging evidence (ie, health apps, artificial intelligence, autonomous decision-support systems)
- Include essential interprofessional competencies (ie, informatics concepts and processes, health information management, communication, ethics, legal, or regulations, and privacy and security)

Target audience or setting to include:

- Allied health professionals
- Low- and middle-income countries

Application:

- Provide case studies of integration into curricula or training programs or examples of application in practice

The findings from this review can also inform and guide future training initiatives on digital health. When designing an educational or training program, it may not be possible to cover numerous competencies presented in a framework; rather, the program should focus on a specific set of competencies suitable for a particular group of health care workers, role, level of seniority, geographical, and health care setting. However, the identified competency domains prevalent in more than half of the included frameworks (ie, informatics concepts and processes, health information management, communication, ethics, legal, or regulations, and privacy and security) should be considered essential interprofessional competencies and thus should be incorporated into training and education efforts for any health care worker group. In addition, several digital competencies presented in the included frameworks may already be covered within the existing curriculum. For example, competencies within the *Analysis* and *Research* category may have been integrated within epidemiology training and evidence-based medicine education, respectively. Therefore, educators should consider integrating digital health training within existing parts of the curriculum and teaching it in an applied manner as much as possible. For example, the use of EHRs could be incorporated into the internal medicine curriculum.

### Strengths and Limitations

To our knowledge, this is the first consolidation and analysis of existing digital health competency frameworks regardless of the role of health care workers. We performed a thorough search, including several databases and gray literature sources. Our analysis also provides a comprehensive overview of the types of competencies presented in digital health competency frameworks, which will aid in the training and education of health care workers to be digitally competent in relevant areas. Some weaknesses must be kept in mind when interpreting the findings of this review. Although a substantial number of frameworks from gray literature were included, some could have been missed, potentially national guidelines and standards from LMICs. In addition, when performing the thematic analysis, there were frameworks with vague competency categories and overlaps among some categories, leading to differences in opinions during the classification process. However, the 2 reviewers used their expertise to develop and clearly define the domains and allocate categories from frameworks into these domains, first independently and then through a consensus discussion, to reduce bias and classify as appropriately as possible. Although the reviewers aimed to make the classification process as transparent and reproducible as possible, it must be noted that there could be alternate ways of interpreting and classifying and that categorization may differ in the future with the new advances in digital health.

### Conclusions

Of the 30 frameworks included in this scoping review, a majority target nurses, originate from high-income countries, and have been developed using an iterative approach. Our analysis of digital health competency frameworks can help inform the development of future digital health training programs for health care workers. Existing frameworks largely focus on the development of basic IT skills, proficiency in managing health-related information and digital communications, and awareness of ethical, legal, privacy, and security implications relating to IT. Future frameworks and training programs need to take into consideration the evolving nature of digital health and have to be able to incorporate upcoming digital trends, such as artificial intelligence and robotics. There is also a need for frameworks focusing on LMICs, medical students, and allied health professionals.

## Appendix

Multimedia Appendix 1 Inclusion and exclusion criteria.

Multimedia Appendix 2 Search strategy for Medical Literature Analysis and Retrieval System Online (MEDLINE).

Multimedia Appendix 3 Keywords used for searching gray literature.

Multimedia Appendix 4 List of organizations whose websites were searched.

Multimedia Appendix 5 Data extraction form.

Multimedia Appendix 6 Details of included studies.

Multimedia Appendix 7 Allocation of competency categories or themes from included frameworks into overarching competency domains.

Multimedia Appendix 8 Competency domains prevalent across 30 digital health competency frameworks.

## Abbreviations

AHIEC: Australian Health Informatics Council
CINAHL: Cumulative Index to Nursing and Allied Health Literature
EHR: electronic health record
EMBASE: Excerpta Medica dataBASE
ERIC: Education Resources Information Centre
HITCOMP: Health Information Technology Competencies
IMIA: International Medical Informatics Association
IT: information technology
LMIC: low- and middle-income country
MEDLINE: Medical Literature Analysis and Retrieval System Online
NHS: National Health Services
TIGER: Technology Informatics Guiding Education Reform
