# Supplementary material for: A Digitally Competent Health Workforce: Scoping Review of Educational Frameworks
Source: J Med Internet Res. 2020 Nov 5;22(11):e22706. doi: 10.2196/22706 (PMC7677019; doi:10.2196/22706)
Supplement: Multimedia Appendix 1 [file jmir_v22i11e22706_app1.docx]

# Appendix 1: Inclusion and exclusion criteria.

**Inclusion criteria**

Content

- Frameworks that identify, structure and list competency categories or competencies on digital technologies and concepts inclusive of the following:

ehealth or e-health or digital health or health technology or health information technology, health informatics, educational technology, electronic medical record (EMR), electronic health record (EHR), electronic patient record, digital health record, email/database/electronic health, telehealth, mhealth, m-health, smartphone applications (apps), telemedicine, telemonitoring, teleradiography or anything that starts with “tele”, computer-assisted or computer-based (i.e. computer-assisted history taking), computerized disease registries, clinical decision support, computerized provider/physician order entry, electronic prescribing (e-prescribing), artificial intelligence (AI), clinical management systems, hospital information system, internet (online), intranet, LAN, web, simulated/simulation learning modality of a virtual or electronic nature, virtual reality/anything involving distance learning, use of “software name”, robot/robotic network, remote, computer-based, CD-ROM, DVD, telemedicine/teleadvice for learning, “streaming”, gaming, gamification, MOOC, digital games/anything, m or mobile device, multimedia, 2-D, 3-D, podcast, video/videotape, ”serious games” as part of digital games, head mounted display/goggles, immersion/immersive experience as part of virtual reality, avatar, computer generated patient, augmented reality, mixed reality, LapSim, Box trainer, 2^nd^ life, general or non-specific games/interactive/training program/workshop with no mention of any “e” or “digital” component.

Participants

- Frameworks should target health workers inclusive of the following:

All pre-service and in-service health workers in medicine, nursing, and allied health (examples include**:** physio/occupational therapists, pharmacists, radiographers, radiotherapists, paramedics, environmental and occupational health and hygiene professionals, audiologists, speech therapists, nutritionists/dieticians, medical /nuclear medicine technologists, optometrists/opticians, public health staff including “”medical/clinical epidemiologists”, “medical/clinical social workers”, “clinical/medical psychologist”), students in these areas, pre-medical students, community health agents, educators/counsellors in any healthcare domain

Timeline

- Frameworks published from year 2000 onwards

**Exclusion criteria**

- Articles published before 1^st^ January 2000
- Frameworks that present competencies on non-digital technology/concepts inclusive of the following:

Physical/real tools, mannequin without digital components, plastic dummy with haptic feedback but does not include “e” or “digital”, hard copies, cassettes, real/live patients used as educational tools, blended learning where all learning components in the intervention are clearly NOT “e” or “digital”, standardized patients using real patients

- Competencies relating to evidence-based practice and information literacy, except when other digital health competencies are also covered within a framework
- Digital health competencies for non-health workers inclusive of the following:

Patients, general population, students, trainees and professionals in professions other than health, trainers/educators with both health and non-health backgrounds, staff/students in “traditional medicine,” alternative medicine“, “complementary medicine”, engineering, Chinese medicine, homeopathy, acupuncturist, herbalist, Ayurvedic, chiropractor, students in basic science including pharmacology, anatomy, naturopath, non-medical/clinical social worker or psychologist (except clinical psychologist) or technologist with no medical/clinical or related term in front

- Conference abstracts, posters and book reviews/opinion pieces/commentaries/viewpoint articles/editorials/letter to Editor
