# Supplementary material for: A Digitally Competent Health Workforce: Scoping Review of Educational Frameworks
Source: J Med Internet Res. 2020 Nov 5;22(11):e22706. doi: 10.2196/22706 (PMC7677019; doi:10.2196/22706)
Supplement: Multimedia Appendix 2 [file jmir_v22i11e22706_app2.docx]

# Appendix 2: Search strategy for Medline.

1 Computer User Training/ or Computer Literacy/ (3328)

2 ((ehealth or e-health or digital health or health technolog* or health information technolog* or informatics or educational technolog* or electronic medical record? or electronic health record? or electronic patient record? or digital health record? or electronic prescribing or eprescribing or telehealth or mhealth or m-health or telemedicine or telemonitoring or clinical decision support system? or teleradiology or telenursing or hospital information system? or computer assisted decision making) adj4 (competenc* or literac* or skill? or capabilit* or capacit* or readiness or curricul*)).ab,ti. (1438)

3 exp Health Personnel/ or exp Students, Health Occupations/ (547308)

4 (health personnel or healthcare profession* or health profession* or healthcare worker? or health worker? or healthcare provider? or health provider? or health service provider? or physician? or doctor? or medic* or premedic? or medical student? or nursing student? or nurse student? or medical graduate? or undergraduate medical or postgraduate medical or health graduate? or resident? or medical practitioner? or general practitioner? or general medicine or family medicine or primary care or nurs* or dentist? or dental or predent* or pharmac*).ab,ti. (3464534)

5 3 or 4 (3677803)

6 2 and 5 (988)

7 1 and 5 (1980)

8 6 or 7 (2861)

9 limit 8 to yr="1990 -Current" (2748)

(Time limit: 1^st^ January 1990 – 6^th^ November 2019)

Number of search results from Medline using this search strategy: 2,748

**Total number of search results across the 6 databases: 14,091**
