# Supplementary material for: A Digitally Competent Health Workforce: Scoping Review of Educational Frameworks
Source: J Med Internet Res. 2020 Nov 5;22(11):e22706. doi: 10.2196/22706 (PMC7677019; doi:10.2196/22706)
Supplement: Multimedia Appendix 3 [file jmir_v22i11e22706_app3.docx]

# Appendix 3: Keywords used for searching gray literature

1. Digital health competency
2. e-health competency
3. e-health literacy
4. eHealth literacy
5. ehealth competency
6. eHealth competency
7. ehealth literacy
8. Digital health literacy
9. Computer literacy
10. Computer user training
11. Health informatics competency
12. Information technology competency
13. Informatics literacy
14. Informatics competency
15. Information literacy need
16. Information literacy skill
17. Biomedical competency
18. Digital health capability
19. e-health capability
20. Health technology competency
21. Health information technology
22. Digital health skill
23. Digital health capacity
24. e-health skill
25. ehealth skill
26. eHealth skill
27. e-health ready
28. ehealth ready
29. eHealth ready
30. Digital health ready
31. Health informatics
32. Clinical informatics
33. Bioinformatics
34. Medical informatics
35. Imaging informatics
36. Biomedical informatics
37. Surgical informatics
38. Clinical competence
39. Competency-based education
