# Supplementary material for: A Digitally Competent Health Workforce: Scoping Review of Educational Frameworks
Source: J Med Internet Res. 2020 Nov 5;22(11):e22706. doi: 10.2196/22706 (PMC7677019; doi:10.2196/22706)
Supplement: Multimedia Appendix 4 [file jmir_v22i11e22706_app4.docx]

# Appendix 4: List of organizations whose websites were searched.

1. Accreditation Council for Graduate Medical Education
2. American Medical Association Graduate Medical Education Competency Education Program
3. American Nursing Informatics Association
4. American Telehealth Association
5. Australian College of Nursing
6. Australian Digital Health Agency
7. Australian Health Informatics Education Council
8. Australian Nursing & Midwifery Federation
9. Canada Health Infoway
10. Digital Health Canada
11. European Federation for Medical Informatics
12. European Medical Students Association
13. GCC eHealth Workforce Development Conference
14. Healthcare Information and Management Systems Society
15. Health Informatics Society of Australia
16. International Medical Informatics Association
17. NHS Digital
18. NHS: Health Education England
19. Pan American Health Organization
20. Royal College of Nursing
