# Supplementary material for: A Digitally Competent Health Workforce: Scoping Review of Educational Frameworks
Source: J Med Internet Res. 2020 Nov 5;22(11):e22706. doi: 10.2196/22706 (PMC7677019; doi:10.2196/22706)
Supplement: Multimedia Appendix 5 [file jmir_v22i11e22706_app5.docx]

# Appendix 5: Data extraction form.

- Study ID (First author and year)
- Title
- Source (hyperlink)
- Methodology used for framework development
- Team/institution involved in framework development
- Geographical setting
- Healthcare setting
- Target health worker population
- Intended purpose (e.g. education or practice)
- Digital health area (e.g. health informatics, nursing informatics, eHealth)
- Describe the structural composition of framework (e.g. classified based on categories, proficiency level, health worker role, etc)
- List competency areas/categories/themes
- Number of competencies covered
- Information on applications to education or practice
- Original framework or adapted from a previously published framework?
- Future plans on updating framework (indicate if any)
