# Supplementary material for: A Digitally Competent Health Workforce: Scoping Review of Educational Frameworks
Source: J Med Internet Res. 2020 Nov 5;22(11):e22706. doi: 10.2196/22706 (PMC7677019; doi:10.2196/22706)
Supplement: Multimedia Appendix 6 [file jmir_v22i11e22706_app6.docx]

# Appendix 6: Details of included studies

| **Article No.** | **Study ID  (First author, year)** | **Title** | **Intended setting  (country; healthcare)** | **Intended audience (profession, educational level)** | **Digital health area** | **Objective(s) of framework** | **Methodology** | **Structural composition of framework** | **Framework developed by:** | **Framework applicability to training initiatives** |
| --- | --- | --- | --- | --- | --- | --- | --- | --- | --- | --- |
| 1 | Academy of Medical Royal Colleges, 2011 [59] | E Health competency framework - defining the role of the expert clinician | Scotland;  All | Doctors and dentists;  Pre-service and in-service | eHealth | To define recognised competencies in eHealth that can be acquired by medical and dental trainees. | Framework aligned with other national competency frameworks. | 418 competencies are grouped into 20 domains, where competencies are sorted based on outcomes for each domain (i.e. knowledge, skills, behaviour). | Academy of Medical Royal Colleges and The Scottish Government | Competencies listed are specific skills, thus able to guide training initiatives. |
| 2 | Australian Health Informatics Education Council, 2011 [57] | Health Informatics: Scope, Careers and Competencies | Australia;  All | All HCPs including admin and IT support staff;  Pre-service and in-service | HI | To identify the scope of HI and the skills required by people working in the healthcare industry. | Literature review drew on a wide range of international and national initiatives to inform the competency development. | 45 competencies are grouped into 3 broad categories (i.e. 1) Knowledge; 2) Medicine, Health and Biosciences, Health System Organisation; 3) Informatics/Computer Sciences, Mathematics, Biometry) and each competency is assigned a competency level ranging from 1 to 6 (i.e. 1) Remembering; 2) Understanding; 3) Applying; 4) Analysing; 5) Evaluating; 6) Creating;) for each role (i.e. 1) Healthcare professionals, 2) Health informatician, 3) Specialist examples based upon existing HI related roles). | Australian Health Informatics Council (AHIEC) | Competencies listed are specific skills, thus able to guide training initiatives. |
| 3 | Association of Faculties of Medicine of Canada in Partnership with Canada Health Infoway, 2014 [55] | eHealth Competencies for Undergraduate Medical Education | Canada;  Not specified | Medical students;  Pre-service | eHealth | To develop eHealth competencies for undergraduate medical education. | Framework was based on contributions from an expert group involving individuals from the Faculties of Medicine across Canada and key stakeholder groups | 25 competencies are classified according to physicians' roles (i.e. 1) Communicator, 2) Collaborator, 3) Scholar, 4) Professional, 5) Health Advocate, 6) Medical Expert, 7) Leader) and each competency is further subdivided into preclinical and clerkship milestones. | The Association of Faculties of Medicine of Canada in Partnership with Canada Health Infoway, | Competencies listed are specific skills, thus able to guide training initiatives. |
| 4 | Australian Nursing and Midwifery Federation, 2015 [42] | National Informatics Standards for Nurses and Midwives | Australia;  All | Nurses & Midwives (registered nurses, midwives and enrolled nurses);  In-service | NI | To develop informatics standards for nurses and midwives which could facilitate the on-going development of a skilled workforce. | 1. Literature review  2. Consensus via focus groups, on-line survey, and interviews with individual nurses and midwives | 53 competencies are grouped into 3 broad categories (i.e. 1) Computer Literacy; 2) Information Literacy; 3) Information Management) and further divided into 10 sub-categories. | Australian Nursing & Midwifery Federation | Competencies listed are specific skills, thus able to guide training initiatives. |
| 5 | Ayres, 2012 [45] | Nutrition Informatics Competencies across All Levels of Practice: A National Delphi Study | US;  All | Registered dietitians, and dietetic technicians, registered and students;  Pre-service and in-service | Nutrition informatics | To establish the first compilation of informatics competencies for registered dietitians (RDs) and dietetic technicians, registered (DTRs) and students. | 1. Literature review 2. Delphi was conducted with practitioners from five constituencies: educators, clinical or community practice, informatics, administration and management, and Academy staff. | 216 competencies are grouped into 3 broad categories (i.e. computer skills, informatics knowledge, informatics skills) and for each level of practice (i.e. Novice; Beginner; Competent; Proficient; Informatics Specialist; Informatics Expert). | Academy’s Nutrition Informatics Committee | Case studies are provided, illustrating the use of competencies. |
| 6 | Barakat, 2013 [50] | eHealth Technology Competencies for Health Professionals Working in Home Care to Support Older Adults to Age in Place: Outcomes of a Two-Day Collaborative Workshop | Netherlands;  Home care | Nurses;  Pre-service and in-service | eHealth | To identify the required competencies for HCPs working with eHealth technologies in home care. | A two-day collaborative workshop was undertaken with academics with experience on the application and development of technologies to support older people. | 14 competencies are organised into 5 themes (i.e. 1) the requirements for basic ICT, proficiency, quantitative analysis, and interpretation skills, 2) communication skills, 3) support and guidance for the patient (both for care support, computer, and ICT use), 4) knowledge of best practices, and 5) legal requirements concerning patient privacy and confidentiality). | University of Sourth Florida | Competencies listed are specific skills, thus able to guide training initiatives. |
| 7 | Brunner, 2018 [47] | An eHealth Capabilities Framework for Graduates and Health Professionals: Mixed-Methods Study | Australia;  Not specified | Health graduates;  Pre-service | eHealth | To inform and modify competency-based frameworks that better reflect the complexity of real-life environments as is found in the field of using eHealth for health care. | 1. Literature review 2. Focus group discussion 3. Delphi study | 40 competencies are organised into 4 broad domains (i.e. Digital Technologies, Systems, and Policies; Clinical practice; Data analysis and knowledge creation; Technology implementation and codesign), and further divided into knowledge and performance cues. | University of Sydney | Competencies listed are specific skills, thus able to guide training initiatives. |
| 8 | Chang, 2011 [21] | Nursing informatics competencies required of nurses in Taiwan | Taiwan;  Not specified | Nurses;  In-service | NI | To provide a master list of NI competency requirements for nurses at four levels of practice in Taiwan. | Three Web-based Delphi rounds were conducted with expert groups in nursing, educators and administrators. | 318 competencies are grouped into 3 broad categories (i.e. computer skills, informatics knowledge, informatics skills) for each level of practice (i.e. beginning nurse, experienced nurse, informatics specialist, informatics innovator). | University of Utah | Competencies listed are specific skills, thus able to guide training initiatives. |
| 9 | Collins, 2017 [46] | Nursing Informatics Competency Assessment for the Nurse Leader: The Delphi Study | Not specified; Not specified | Nurses leaders;  In-service | NI | To provide relevant NI competencies for curricula design and development for nurse executives, leaders, and nurse managers. | 1. Literature review 2. Delphi study (3 rounds) 3. Exploratory factor analysis for scale optimization and factor identification | 74 competencies are organised into 15 categories. Also, 15 out of the 74 competencies are ranked by priority. | Harvard Medical School, Boston, Massachusetts | The most relevant competencies are ranked, thus able to guide training initiatives. |
| 10 | Crawford, 2016 [51] | Defining competencies for the practice of telepsychiatry through an assessment of resident learning needs | Not specified; Remote care | Psychiatry residents;  In-service | Telepsychiatry | To guide the development of telepsychiatry curricula in postgraduate psychiatry training. | Expert panel and interviews with faculty and psychiatry residences. | 15 competencies are classified by professional roles (i.e. 1) Medical expert, 2) Communicator, 3) Collaborator, 4) Manager, 5) Advocate) and example quotes are presented for competencies. | University of Toronto, Canada; Centre for Addiction and Mental Health, Toronto, Canada | Examples are provided to guide the use of competencies. |
| 11 | Curran, 2003 [22] | Informatics competencies for nurse practitioners | Not specified; Not specified | Nurse practitioners;  Pre-service and in-service | NI | To propose a list of NI competencies essential for nurse practitioner education and practice. | A small team of nurse informaticists and nurse practitioner program directors discussed and agreed on the competencies | 32 competencies are grouped into 3 broad categories (i.e. computer skills, informatics knowledge, informatics skills). | Columbia University, School of Nursing | Competencies listed are specific skills, thus able to guide training initiatives. |
| 12 | Egbert, 2016 [33] | An iterative methodology for developing national recommendations for nursing informatics curricula | Austria, Switzerland, Germany;  Not specified | Nurses;  Pre-service | NI | To develop national recommendations for NI competencies and demonstrate how they can be utilized practically in NI curricula development. | 1. Literature review 2. Survey 3. Focus group discussion to finalise competencies. | 24 competency areas are identified, 6 of the most relevant areas are ranked for 5 nursing roles (i.e. 1) Nursing Management; 2) IT Management; 3) Quality Management; 4) Clinical Nursing; 5) Inter-professional Coordination of Care) in Austria, Germany, and Switzerland collectively. | Osnabrück University, Germany | Case studies are provided, where the framework findings have been integrated into universities' curriculum; The most relevant competencies are ranked, thus able to guide training initiatives. |
| 13 | Egbert, 2019 [34] | Competencies for nursing in a digital world. Methodology, results, and use of the DACH-recommendations for nursing informatics core competency areas in Austria, Germany, and Switzerland |  |  |  |  |  | 24 competency areas are identified, 5 of the most relevant areas are ranked for 5 nursing roles for each country (i.e. Austria, Germany, and Switzerland). | Osnabrück University, Germany |  |
| 14 | HITCOMP, 2019 [41] | Health Information Technology Competencies | International; Acute care | All HCPs;  Pre-service and in-service | HI | To provide a framework to measure, inform, educate and advance eHealth skills, education and knowledge among HCPs. | 1. Literature review 2. Survey was sent to experts from multiple countries.  3. Gap analysis was conducted to identify gaps in eHealth areas.  4. Expert consultation | 1025 competencies are organised into 33 competency areas, for 5 levels of practice (i.e. Baseline; Basic; Advanced; Intermediate; Expert) across 5 domains (i.e. Administration; Direct Patient Care; Engineering/Information Systems/ICT; Informatics; Research/Biomedicine). | EU-US eHealth collaboration Workforce Development Workgroup., 2019 | Competencies listed are specific skills, thus able to guide training initiatives. |
| 15 | Hilty, 2015 [60] | A framework for telepsychiatric training and e-health: competency-based education, evaluation and implications | Not specified; Remote care | Doctors, medical students;  Pre-service and in-service | Telepsychiatry and eHealth | To serve as a guide for the implementation and evaluation of telepsychiatry training and assessment methods. | Competencies are organized using the US Accreditation Council of Graduate Medical Education framework, with input from the CanMEDS framework. | Competencies are listed for 8 categories (i.e. 1) patient care; 2) communications; 3) systems-based practice; 4) team work; 5) professionalism; 6) practice-based learning; 7) knowledge; 8) technology), which are further sorted into sub-categories, for each level of practice (i.e. novice or advanced beginner; competent/proficient; expert). | Collaborative effort with US and Canada universities and institutions. | Competencies listed are specific skills, thus able to guide training initiatives. |
| 16 | Honey, 2018 [58] | Guidelines: Informatics for Nurses Entering Practice | New Zealand; Not specified | Registered nurses;  Pre-service | NI | To provide beginning registered nurses guidelines on NI that form a bridge between theory, education and practice. | 1. Curriculum mapping highlighted a mismatch between nursing education and industry requirements. 2. Literature review to finalise competencies. | 4 broad domains are identified (i.e. Professional Practice; Information Management; Information and communication technologies to enhance the health of New Zealanders; General computer and ICT Skills), where relevant subcategories and examples are presented. | Collaborative effort with New Zealand institutions, health boards and councils. | Examples are provided to guide the use of competencies. |
| 17 | Hubner, 2016 [35] | Towards an international framework for recommendations of core competencies in nursing and inter-professional informatics: The TIGER competency synthesis project | International; Not specified | Nurses;  Pre-service | HI | To empirically define and validate a framework of globally accepted core competency areas in HI and to enrich this framework with exemplar information derived from local educational settings. | 1. Survey was sent to experts from multiple countries. 2. A workshop was held to validate competencies. | 24 competency areas are identified, and 6 of the most relevant areas are ranked for 5 nursing roles (i.e. 1) Nursing Management; 2) IT Management; 3) Quality Management; 4) Clinical Nursing; 5) Inter-professional Coordination of Care). | University Applied Sciences Osnabrück, Health Informatics Research Group, Germany | Case studies are provided, illustrating the use of competency areas; The most relevant competencies are ranked, thus able to guide training initiatives. |
| 18 | Hubner, 2018 [36] | Technology Informatics Guiding Education Reform - TIGER |  |  |  |  |  | 24 competency areas are identified, and 10 of the most relevant areas are ranked for 5 nursing roles. The 24 core competency areas are also sorted into 6 overarching domains (i.e. Data, information and knowledge; Information exchange and information sharing; Ethics and legal issues; Systems life cycle management; Management in informatics; Biostatistics and medical technology). |  |  |
| 19 | Hubner, 2019 [52] | Towards the TIGER International Framework for Recommendations of Core Competencies in Health Informatics 2.0: Extending the Scope and the Roles | International; Not specified | All HCPs;  Pre-service | HI | To extend the scope of the framework of recommendations of HI core competencies areas beyond nursing and to include other HCP roles. | 1. Expert panel drew on the competency areas identified by Hubner (2016 and 2018) and revised the areas accordingly to include more HCP roles.  2. Survey was sent to experts from multiple countries | 33 competency areas are identified, the 10 most relevant areas are ranked for each HCP role (i.e.1) Direct patient care (nurses/physicians/therapists); 2) Health information management; 3) Executives (clinical and administrative); 4) Chief information officers (clinical and technical); 5) Engineering or health IT specialist; 6) Science and education). | EU*US eHealth Work project | Case studies are provided, illustrating the use of competency areas; The most relevant competencies are ranked, thus able to guide training initiatives. |
| 20 | Hwang, 2008 [48] | A study of the informatics literacy of clinical nurses in Taiwan | Taiwan; Hospital | Clinical nurses;  In-service | Informatics literacy | To identify informatics literacy requirements of clinical nurses for the integration of knowledge and skills into nursing education curricula. | 1. Literature review 2. Survey on identified competency items was sent to nursing participants. | 49 competencies are grouped into 3 broad categories (i.e. computer attitudes, informatics knowledge, informatics skills), and further grouped into sub-categories. | Collaborative effort with Taiwan universities and institutions. | Competencies listed are specific skills, thus able to guide training initiatives. |
| 21 | Jidkov, 2019 [40] | Health informatics competencies in postgraduate medical education and training in the UK: a mixed methods study | UK;  Not specified | Doctors;  In-service | HI | To establish key HI competencies for postgraduate medical education to be integrated into training curricula. | 1. Literature review 2. Curricular content analysis  3. Expert consultation | 20 competencies were organised into 6 domains (i.e. Information Governance & Security; System Use and Clinician Safety; Digital Communication; Information and Knowledge Management; Patient Empowerment; Emerging Technologies). | Collaborative effort with UK universities and institutions. | Only competency domains and brief descriptions are provided. |
| 22 | Maheu, 2018 [39] | Correction to: An Interprofessional Framework for Telebehavioral Health Competencies | Not specified; Remote care | All HCPs including allied health professionals;  Pre-service and in-service | Telebehaviour | To develop telebehavioural competencies for practitioners, trainers, graduate students, and supervisors to address the disparities between behavioural disciplines. | 1. Literature review 2. Expert consultation (leaders from a variety of professional organizations) | 7 broad domains of expertise are identified, which are further broken down into 51 telebehavioral objectives, followed by 149 telebehavioural practices across 3 levels (i.e. Novice, Proficient & Authority). | Collaborative effort with US universities and institutions. | Competencies listed are specific skills, thus able to guide training initiatives. |
| 23 | Mantas, 2010 [49] | Recommendations of the International Medical Informatics Association (IMIA) on Education in Biomedical and Health Informatics. First Revision | International, Not specified | All HCPs;  In-service | BMHI | To recommend learning outcomes for healthcare professionals either as IT users or as BMHI specialists and to support international initiatives concerning education in BMHI. | Recommendations were discussed and refined by IMIA task force. | 34 competencies are organised into 3 BMHI domains (i.e. (1) Biomedical and Health Informatics Core Knowledge and Skills; (2) Medicine, Health and Biosciences, Health System Organization; (3) Optional Modules in BHMI and from Related Fields) and further sorted according to 3 levels of proficiency (i.e. introductory, intermediate, advanced), and each competency is determined if its required by a IT user or BMHI specialist according to 3 levels (i.e.. introductory; intermediate; advanced). | IMIA | Competencies listed are specific skills, thus able to guide training initiatives. |
| 24 | Nagle, 2014 [37] | Developing entry-to-practice nursing informatics competencies for registered nurses | Canada;  Not specified | Registered nurses;  Pre-service | NI | To describe the outcomes of informatics entry-to-practice competencies for adoption by Canadian Schools of Nursing. | 1. Literature review  2. Consensus with experts through three rounds of feedback | 19 competencies are sorted according to 3 overarching domains (i.e. 1) Uses relevant information and knowledge to support the delivery of evidence-informed patient/client care, 2) Uses ICTs in accordance with professional and regulatory standards and workplace policies, 3) Uses ICTs in the delivery of patient/client care). | The Canadian Association of Schools of Nursing (CASN) | Competencies listed are specific skills, thus able to guide training initiatives. |
| 25 | NHS, 2018 [56] | A Health and Care Digital Capabilities Framework | UK;  All | All HCPs;  In-service | Digital literacy | To provide a guiding framework for HCPs to extend their digital capabilities. | Consultations with different stakeholders and workforce groups [62] | An overarching domain (i.e. digital identity, wellbeing, safety and security) is broken down into 5 domains (i.e. communication, collaboration and participation; teaching, learning and self-development; information, data and content literacies; creation, innovation and research; technical proficiency), where each domain has a domain description followed by specific capabilities sorted according to 4 proficiency levels. | NHS | Competencies listed are specific skills, thus able to guide training initiatives. |
| 26 | Public Health Informatics Institute, 2016 [38] | Applied Public Health Informatics Competency Model | US;  Not specified | Public health professionals;  In-service | Informatics | To provide a working framework that serves as public health informatics competency model. | 1. Literature review (synthesized existing competencies from frameworks on Public Health Professionals and Informaticians) 2. Expert consultation | 8 categories were identified (i.e. Principles and Strategy; Standards and Interoperability; Project Management; Information Systems; Communication; Evaluation; Analysis, Visualization and Reporting; Policy), and for each an overarching competency statement and pertaining competencies are listed. | Public Health Informatic Institute | Competencies listed are specific skills, thus able to guide training initiatives. |
| 27 | JASEHN, 2018 [61] | Recommendations on a Common Framework for Mapping Health Professionals ’ eHealth Competencies | Region;  Not specified | All HCPs including admin and IT support staff;  In-service | eHealth | To propose a conceptual framework for eHealth profiles and competencies, aiming to address all relevant tasks included in eHealth service’s lifecycle. | 1. Literature review  2. Framework aligned with roles and competences as per the European eCompetence Framework  3. Use of framework descriptions to determine skill level for each role [63] | 2 models are presented.  Model 1: Mission and main tasks were described for 3 main profiles of workers in health settings (i.e. health, non-health and IT).  Model 2: 52 competencies were grouped into 6 domain areas (i.e. manage; plan; build; use; run; enable), where each competency is provided with a description, mapped according to 5 proficiency levels, with a set of associated knowledge and skill. | Joint Action to Support the eHealth Network (JASEHN) | Competencies listed are specific skills, thus able to guide training initiatives. |
| 28 | Staggers, 2001 [31] | Informatics competencies for nurses at four levels of practice | Not specified; Not specified | Nurses;  In-service | NI | To determine comprehensive NI competencies by level of nursing practice, for developing nursing and health curricula and job descriptions. | 1. Literature review 2. Consensus with expert panel | 304 competencies are grouped into 3 categories (i.e. computer skills; informatics knowledge; informatics skills) for each level of practice (i.e. beginning nurse; experienced nurse; informatics specialist; informatics innovator). | University of Utah College of Nursing | Competencies listed are specific skills, thus able to guide training initiatives. |
| 29 | Staggers, 2002 [32] | A Delphi study to determine informatics competencies for nurses at four levels of practice |  |  |  |  | Three Delphi rounds were conducted. | 281 competencies are grouped into 3 broad categories (i.e. computer skills, informatics knowledge, informatics skills) for each level of practice (i.e. beginning nurse, experienced nurse, informatics specialist, informatics innovator). | Clinical Informatics, University of Utah; Bureau of Health Professions, Health Resources and Services Administration; Ohio State University |  |
| 30 | Thye, 2018 [54] | What Are Inter-Professional eHealth Competencies? | International; Not specified | All HCPs including admin and IT support staff;  Pre-service | eHealth | To identify competencies which are at the intersection of the individual groups of HCPs. | 1. Mapped competency areas from Hubner (2018) and HITCOMP.  2. Survey was sent to HCPs. | 33 competency areas are identified, and the 10 most relevant interprofessional areas are identified for the following HCP roles: 1) Direct patient care (physicians, nurses, pharmacists, other HCPs); 2) Executives (technical and clinical CEOs / CIOs); 3) IT (engineering / IT specialist); and 4) Science & education. | Collaborative effort with US and Germany institutions. | The most relevant competencies are ranked, thus able to guide training initiatives. |
| 31 | Trangenstein, 2009 [43] | Nursing informatics for future nurse scholars: lessons learned with the doctorate of nursing practice (DNP) | Not specified; Not specified | Nurse scholars;  In-service | NI | To differentiate the NI knowledge and skills required by 3 different levels of practice. | 1. Literature review 2. Discussion to come up with a model | 7 competency domains sorted according to nursing level of practice (i.e. Entry Level Nurse; Advanced Practice Nurse; Nurse Scholar). | Frist Nursing Informatics Center, School of Nursing, Vanderbilt University | Only broad competency domains are provided. |
| 32 | Van Houwelingen, 2016 [53] | Competencies required for nursing telehealth activities: A Delphi-study | Not specified; Remote care | Nurses;  Pre-service | Telehealth | To identify competencies for nursing telehealth education. | 1. Survey with relevant competencies was sent to participants. 2. Delphi study (4 rounds) | 52 competencies are organised into 3 broad categories (i.e. knowledge; attitude; skills), where skills are further subcategorised into 5 skills (i.e. general; technological; clinical; communication; implementation). | Research Center for Innovations in Health Care, Faculty of Health Care, Utrecht University of Applied Sciences, The Netherlands | Competencies listed are specific skills, thus able to guide training initiatives. |
| 33 | Westra and Delaney, 2008 [44] | Informatics competencies for nursing and healthcare leaders | Not specified, Not specified | Nurse leaders;  In-service | NI | To identify NI competencies that are unique to the role of the nursing leader. | 1. Literature Review 2. Delphi | 92 competency areas are grouped into 3 broad categories (i.e. computer skills; informatics knowledge; informatics skills). | University of Minnesota, School of Nursing, Minneapolis | Competencies listed are specific skills, thus able to guide training initiatives. |

HCP, Healthcare professional; HI, Health informatics; NI, Nursing informatics; BMHI, Biomedical and health informatics; ICT, Information and communications technology.
