# Supplementary material for: A Digitally Competent Health Workforce: Scoping Review of Educational Frameworks
Source: J Med Internet Res. 2020 Nov 5;22(11):e22706. doi: 10.2196/22706 (PMC7677019; doi:10.2196/22706)
Supplement: Multimedia Appendix 7 [file jmir_v22i11e22706_app7.docx]

# Appendix 7: Allocation of competency categories and themes from included frameworks into overarching competency domains

| **Domains** | **Frequency** | **Article No.* & Categories/Themes/Statements** |
| --- | --- | --- |
| Administration and general management | 12 | 1. Healthcare planning and delivery & 1. Managing service and performance // 8. Administration // 12. and 13. Resource planning and logistics & 12. and 13. Human resource management in nursing informatics // 14. Administration/General Management/Governance & 14. Business Process Design/Workflows // 15. Administration // 17 and 18. Resource planning and logistics & 17 and 18. Human resource management // 19. Resource planning & management // 22. Administrative Procedures // 28 and 29. Administration // 30. resource planning and management // 10. Administrative abilities // 23. Socio-organizational and socio-technical issues, including workflow/process modelling and reorganization // |
| Analysis | 14 | 6. the requirements for basic ICT, proficiency, quantitative analysis, and interpretation skills **/**/ 7. Data analysis and knowledge creation // 8. Analysis & 8. Evaluation // 12. and 13. Biostatistics/statistics // 14. Data Compiling, Analysis, Modelling and Reporting. // 17 and 18. Biostatistics/statistics // 19. Data analytics // 26. Analysis, Visualization and Reporting (AVR) & 26. Evaluation // 28 and 29. Analysis // 33. Analysis/Evaluation // 30. data analytics // 23. Principles of data representation and data analysis using primary and secondary data sources, principles of data mining, data warehouses, knowledge management // 11. Uses data and statistical analyses to describe and evaluate practice // 24. Analyses, interprets, and documents pertinent nursing data and patient data using standardized nursing and other clinical terminologies (e.g., ICNP, C-HOBIC, and SNOMED-CT, etc.) to support clinical decision making and nursing practice improvements // |
| Attitudes toward IT | 4 | 15. Attitude & 15. Cultural, diversity and social determinants of health) & Language/interpreter ability // 20. Behaviour when using IT & Attitude toward applying IT // 10. Demonstrate awareness of/sensitivity to local cultures, beliefs, knowledge systems, resources, healing practices, and views of technology) // 16. Principles of cultural safety and Te Titiri o Waitangi are applied to information management // |
| Clinical care delivery | 15 | 1. Electronic test requesting and results reporting // 4. Understands the concepts, scope and practice of a variety of information and communication technologies that support health and aged care & 4. Demonstrates the ability to use information communication technologies to promote safe, effective use of information to support nursing and midwifery practice // 7. clinical practice and applications // 8. Practice // 12. and 13. Assisting technologies // 15. History taking, Assessment and physical examination, management and treatment planning // 17 and 18. Assistive technology for ageing people // 19. Assistive technology // 22. Clinical Evaluation and Care // 30. assistive technology for ageing people // 32. Clinical // 16. Nurses effectively use ICT to assist with the delivery of quality nursing care to improve patient outcomes // 23. Basic concepts and applications of ubiquitous computing (e.g. pervasive, sensor-based and ambient technologies in health care, health enabling technologies, ubiquitous health systems and ambient assisted-living) // 11. Describes general applications, systems to support clinical care // 24. Demonstrates that professional judgement must prevail in the presence of technologies designed to support clinical assessments, interventions, and evaluation (e.g., monitoring devices, decision support tools, etc.). // |
| Communication | 19 | 6. Communication skills // 8. Communication // 9. Electronic communications // 15. Communication & IPSC, interpersonal skills and communication) // 19. Communication // 21. Digital communication // 25. Communication, collaboration, and participation // 26. Communication // 28 and 29. Communication // 30. Communication // 32. Communication // 33. Electronic Communications // 27. Communication & Integrated Healthcare ICT solutions // 3. The emphasis is on communication for clarity and enhancement of the doctor–patient relationship. // 16. Interpersonal and interprofessional communication using digital mediums. // 23. Use of personal application software for documentation, personal communication including Internet access, for publication and basic statistics // 11. Uses interactive communication devices with patients and other healthcare providers // 2. Use of personal application software for documentation and communication including internet for access to publications and basic statistics // 24. Advocates for the use of current and innovative information and communication technologies that support the delivery of safe, quality care. // |
| Decision support | 12 | 4. Demonstrates collection, use and management of data and information to support decision-making in practice // 12 and 13. Decision support systems // 14. Clinical Decision Support and Pathways. // 17 and 18. Decision support by IT // 19. Clinical decision support by IT // 28 and 29. Decision support // 30. clinical decision support by IT // 27. Health Decision Support Solutions Usage // 3. These competencies focus on how information technologies can enhance an individual’s medical expertise, recognizing that data and telehealth tools are adjuncts to support decision making while medical expertise is vital to management choices. // 23. Methods for decision support and their application to patient management, acquisition, representation, and engineering of medical knowledge; construction and use of clinical pathways and guidelines // 11. Uses decision support systems, expert systems, and aids for differential diagnosis // 24. Uses decision support tools (e.g. clinical alerts and reminders, critical pathways, web-based clinical practice guidelines, etc.) to assist clinical judgment and safe patient care. // |
| Documentation | 13 | 8. Documentation // 12 and 13. Nursing documentation // 14. Documentation process & 14. Coding and Terminologies // 15. Documentation // 17 and 18. Nursing documentation (including terminologies) // 19. Documentation // 22. Documentation // 28 and 29. Documentation // 30. Documentation // 3. These competencies focus on documentation, use and exchange of data, and communication with patients, families, caregivers and colleagues beginning with the individual patient-physician encounter and moving to the systems perspective. // 16. Complete, accurate and relevant data collection and documentation. // 23. Appropriate documentation and health data management principles including ability to use health and medical coding systems, construction of health and medical coding systems // 2. Use of personal application software for documentation and communication including internet for access to publications and basic statistics // |
| Education and training | 12 | 8. Education & 8. Training & 8. Simulation // 12 and 13. Information management in education and continuing education // 15. inter-professional education // 17 and 18. Information management in teaching, training and education // 19. Learning techniques & 19. Teaching, training, education // 25. Teaching, learning and self-development // 28 and 29. Education and Training & 28 and 29. Simulation // 30. teaching, training and education & 30. teaching, training and education & 30. learning technique // 33. Staff education // 3. These competencies focus on the obligation of the individual to sustain and continually improve information and communication technologies best practices to enhance patient care, participate in lifelong learning and teaching, and engage in continual quality improvement and excellence in their own practices. There could be some overlap with medical expert and professionalism. // 16. Use of specific platforms within the educational environment to develop transferrable skills // 11. Evaluates computer-assisted instruction (CAI) as a teaching tool // |
| Ethics, legal, or regulations | 18 | 4. Complies with the legal and regulatory requirements, and ethical principles, for all uses of information and communication technologies used in nursing and midwifery practice // 8. Regulations // 12 and 13. Ethics and IT // 17 and 18. Ethics and legal issues // 19. Legal issues in IT & 19. Ethics in health IT // 6. Legal requirements concerning patient privacy and confidentiality // 9. Ethical/legal concepts // 14. Legal // 15. Licensure regulations as applied to telemedicine care model (medico-legal issues) & 15. Integrity and ethical behaviour // 22. Legal & Regulatory Issues & 22. Evidence-Based & Ethical Practice (with a Social Media subdomain) // 28 and 29. Regulations // 30. ethics in health IT & 30. legal issues in health IT // 33. Ethical/Legal concepts // 10. Adhere to legislation and professional regulatory standards // 16. Nursing Council regulatory obligations // 23. Ethical and security issues including accountability of health care providers and managers and BMHI specialists and the confidentiality, privacy and security of patient data // 11. Applies the principles of data integrity, professional ethics and legal requirements for patient confidentiality and data security // 24. Complies with legal and regulatory requirements, ethical standards, and organizational policies and procedures (e.g. protection of health information, privacy, and security). // |
| Financial management | 10 | 8. Fiscal management // 9. Financial // 12. and 13. Financial management in NI // 14. Financial and Account Management // 15. Patent care (Billing) // 17 and 18. Financial management // 19. Financial management // 28 and 29. Fiscal management & 28 and 29. Research (funding) // 30. financial management // 33. Financial // |
| Health information and records management | 19 | 1. Health records & 1. Collection and storage of health information & 1. Using and Sharing health information // 4. Information literacy cat & 4. Demonstrates understanding of the purpose, basic structures, use and storage of electronic health records // 8. Data and 8. Data access and 8. Data structure and 8. Information literacy // 9. Data issues // 12. and 13. Information and knowledge management in patient care // 14. Access to Information, Protected Health Information, and Health Information Management & 14. Collection of Data/Knowledge Management (Library) & 14. Records management // 17. and 18. Information and knowledge management in patient care // 19. Information and knowledge management in patient care // 21. Information & Knowledge Management // 25. Information, data and content literacies // 28 and 29. Data access & 28 and 29. Data structure // 33. Access Data/Information & 33. Data Issues // 27. Electronic records management & 27. Electronic health information collection & storage // 3. These competencies focus on documentation, use and exchange of data, and communication with patients, families, caregivers and colleagues beginning with the individual patient-physician encounter and moving to the systems perspective. // 16. Information and digital literacy // 23. Need for systematic information processing in health care, benefits and constraints of information technology in health care // 11. Accesses shared data sets // 2. Efficient and responsible use of information processing tools, to support health care practice and decision making // 24. Performs search and critical appraisal of on-line literature and resources (e.g., scholarly articles, websites, and other appropriate resources) to support clinical judgement, and evidence-informed decision making. // |
| Health care quality and safety | 12 | 1. Improving services // 4. Demonstrates the ability to include research, evidence-based practice and quality improvements in supporting the use of information and communication technologies // 8. Monitoring & 8. Quality improvement // 12 and 13. Quality assurance and management // 14. Quality and safety // 15. QI Teaching and learning // 17 and 18. Quality management // 19. Quality and safety management // 28 and 29. Quality improvement & 28 and 29. Monitoring // 30. quality and safety management // 3. These competencies focus on the obligation of the individual to sustain and continually improve information and communication technologies best practices to enhance patient care, participate in lifelong learning and teaching, and engage in continual quality improvement and excellence in their own practices. There could be some overlap with medical expert and professionalism. // 11. Uses applications to aggregate and analyse data for forecasting, accreditation, clinician value, nurse-sensitive outcomes, evidence-based practice, and quality improvement // |
| Imaging | 6 | 1. Digital Imaging // 12 and 13. Image and biosignal processing // 17 and 18. Biomedical imaging and signal processing // 19. medical technology (imaging) // 30. medical technology (imaging) // 23. Biomedical imaging and signal processing // |
| Informatics concepts and processes | 22 | 4. Demonstrates knowledge and skills in computer basics // 6. The requirements for basic ICT, proficiency, quantitative analysis, and interpretation skills & 6. Knowledge of best practices // 8. Basic Desktop software and 8. Impact // 9. Information systems concepts & 9. Technical knowledge // 12. and 13. Information and communication systems for nursing & 12 and 13. Principles of nursing informatics // 14. General HIT knowledge/system use & 14. Information and Communications Technology/Information Systems/IT // 15. Adapt to technology // 17 and 18. Principles of management & 17 and 18. Principles of nursing informatics & 17 and 18. Information and communication systems // 19. Medical technology // 20. Concepts of basic IT & 20. Knowledge of using HIS & 20. The ability to operate computers & 20. The ability to use IT & 20. Network basics // 21. Systems Use & Clinical Safety & 21. Emerging Technologies // 22. Technology // 26. Information Systems // 28 and 29. Basic desktop software & 28 and 29. Impact // 30. principles of health informatics & medical technology // 32. General & 32. Technological // 33. Information Systems Concepts // 16. Knowledge of patient acuity systems, patient management systems, and what patient data can be utilised for, e.g. Care Capacity Demand Management (CCDM). // 23. Characteristics, functionalities and examples of information systems in health care (e.g. clinical information systems, primary care information systems, etc.) // 11. Discusses the impact of computerized information management on the role of the nurse // 2. Evolution of informatics as a discipline and as a profession // 24. Describes the various components of health information systems (e.g., results reporting, computerized provider order entry, clinical documentation, electronic Medication Administration Records, etc.). // |
| Integration and interoperability | 13 | 1. Integrated Healthcare // 9. Collaboration & 9. Standardization // 14. Care Coordination & 14. HIE/Interoperability/Interfaces/Integration // 15. IPE & IPSC (inter-professional education; IPSC, interpersonal skills and communication) domain // 19. Care processes and IT integration & 19. Interoperability and integration / 26. Standards and Interoperability // 25. Communication, collaboration and participation // 30. healthcare processes and IT integration & 30. interoperability and integration // 27. Communication & Integrated Healthcare ICT solutions // 10. Work with interprofessional providers across distance // 3. We believe it is important to jointly develop, advance and use electronic information and management tools, processes and resources to shape health information systems and improve our current ehealth environment through interprofessional, interdisciplinary and stakeholder collaboration. // 16. Shows awareness of nursing as part of an interprofessional team. // 23. Architectures of information systems in health care; approaches and standards for communication and cooperation and for interfacing and integration of component, architectural paradigms (e.g. service-oriented architectures) // 24. Articulates the significance of information standards (i.e. messaging standards and standardized clinical terminologies) necessary for interoperable electronic health records across the healthcare system. // |
| IT advocacy | 5 | 8. Roles (Change agent, liaison) // 15. Systems-based practice (Outreach to community) // 10. Promote and respond to the health needs of individual patients, families, and communities. // 3. These competencies focus on promoting physician advocacy with respect to humanism in the virtual health care workplace, while at the same time maintaining awareness of the competing priorities between the patient’s and health systems’ needs. // 11. Acts as an advocate of system users including patients and colleagues // |
| Leadership and executive management | 14 | 1. Clinical Leadership and Team working & 1. Setting direction // 8. Management // 9. Executive leadership & 9. Management concepts // 12 and 13. Strategic management and leadership & 12. and 13. Change and stakeholder management & 12. and 13. Principles of management & 12. and 13. Process management // 14. Communication and Change Management & 14. Administration/General Mgmt/Governance // 17 and 18. Strategic management and leadership & 17 and 18. Change management and stakeholder management & 17 and 18. Process management // 19. Leadership & 19. Change/stakeholder management & 19. Strategic management & 19. Principles of management & 19. Process management // 26. Principles and Strategy // 28 and 29. Management // 30. Process management & 30. Strategic management & 30. Principles of management & 30. Change and stakeholder management & leadership // 33. Management Concepts // 10. Use technology to address health human resource problems and systemic inequities in access to care // 3. The competencies in this section focus on the clinician’s leadership abilities in clinical service delivery, including the responsibility for ensuring factual and accurate output from electronic health records. // 23. Methods of project management and change management (i.e. project planning, resource management, team management, conflict management, collaboration, and motivation, change theories, change strategies) // |
| Medicines management | 2 | 1. Medicines Management // 14. Medications and Allergies & 14. Order Entry // |
| Patient access and engagement | 13 | 1. Patient access to health information // 6. support and guidance for the patient (both for care support, computer, and ICT use) // 9. Patient-related applications // 14. Patient Centered Interactions/Patient Identification & 14. Patient Access and Engagement/PHRs // 19. Consumer health informatics // 21. Patient Empowerment // 33. Patient Related Applications // 30. consumer health informatics (including patient access and engagement and PHRs) // 27. Patient access & engagement assistance to ICT usage // 16. Supporting patient’s health knowledge: Assists the patient to access, retrieve and evaluate electronic information. // 23. Characteristics, functionalities, and examples of information systems to support patients and the public (e.g. patient-oriented information system architectures and applications, personal health records, sensor-enhanced information systems) // 11. Assists patients to use databases to make informed decisions* // 24. Assists patients and their families to access, review and evaluate information they retrieve using ICTs (i.e. current, credible, and relevant) and with leveraging ICTs to manage their health (e.g. social media sites, smart phone applications, online support groups, etc.). // |
| Privacy and security | 17 | 1. Safe care of clinical information // 8. Privacy/Security // 12 and 13. Data protection and security // 14. Confidentiality/Protected Health Information/Records Management & 14. Privacy and Security // 15. Privacy and confidentiality (medico-legal issues) // 17 and 18. Data protection and security // 19. Data protection and security // 20. Information security // 21. Information Governance and Security // 25. digital identity, wellbeing, safety and security // 28 and 29. Privacy/security // 30. data protection and security // 3. These competencies focus on defining professional boundaries, obligations and responsibilities as they translate into ehealth practices: Knowing that electronic information and communication strategies have the potential to alter the quality of doctor-patient relationship and obscure the limits governing breach of information. // 23. Ethical and security issues including accountability of health care providers and managers and BMHI specialists and the confidentiality, privacy and security of patient data // 11. Describes ways to protect data* // 16. Safe and accurate use of ICT // // 24. Complies with legal and regulatory requirements, ethical standards, and organizational policies and procedures (e.g. protection of health information, privacy, and security). // |
| Project management | 10 | 1. Project Leadership // 8. PM // 12 and 13. PM // 14. Issue Management and Resolution & 14. Project/Program Management. // 17 and 18. PM // 19. PM // 26. PM // 28 and 29. PM // 30. project management // 23. Methods of project management and change management (i.e. project planning, resource management, team management, conflict management, collaboration and motivation, change theories, change strategies) // |
| Public health | 5 | 14. Population Management/Public Health // 19. Public health informatics // 30. public health informatics // 3. This section addresses the need to balance the use of health information for global initiatives while respecting the privacy of individual patient information. It speaks to meaningful use of electronic resources to inform population health strategies. // 23. Public health informatics // |
| Remote care | 10 | 1. Telehealthcare // 12 and 13. eHealth, telematics, telehealth // 14. eHealth, telematics, telehealth // 17 and 18. eHealth, telematics, telehealth // 19. eHealth, telematics, telehealth // 30. ehealth, mhealth, telematics and telehealth // 15. framework focused on telehealth // 22. framework focused on telehealth // 10. Adapt the assessment process to be carried out at a distance // 23. Methods and approaches to regional networking and shared care (eHealth, health telematics applications and inter-organizational information exchange) // |
| Research | 11 | 8. Research // 12 and 13. Information management in research // 14. Research/Biomed // 17 and 18. Information management in research // 19. Information management research // 25. Creation, innovation and research // 28 and 29. Research // 30. information management in research & 30. information management in research // 33. Clinical research // 23. Information literacy: library classification and systematic health related terminologies and their coding, literature retrieval methods, research methods and research paradigms // 11. Describes general applications available for research // |
| Risk management | 6 | 12 and 13. IT risk management // 14. Risk and Compliance // 17 and 18. IT risk management // 19. IT RM // 30. IT risk management // 23. Handling of the information system life cycle: analysis, requirement specification, implementation and/or selection of information systems, risk management, user training // |
| Systems implementation | 9 | 1. Sourcing, developing and implementing healthcare information and communication technology systems and applications // 7. system and technology implementation // 8. Implementation // 9. Implementation/management // 14. Systems Development and Implementation // 19. System lifecycle management // 28 and 29. Systems Development and Implementation // 32. Implementation // 33. Implementation/ Management // |
| Technical knowledge and support | 15 | 1. Maintenance and support of healthcare IT systems & 1. Infrastructure and Technologies & 1. Clinical IT systems // 8. Systems selection & 8. Systems & 8. Systems maintenance & 8. Testing & 8. Requirements & 8. Programming // 9. Advanced software applications // & 12. and 13. Applied computer science // 14. Informatics Process // 17 and 18. Applied computer science/informatics // 19. ICT/systems (applications) & 19. ICT/systems (architectures) & 19. Applied computer science // 25. Technical proficiency // 28 and 29. Systems & 28 and 29. Systems selection & 28 and 29. Programming & 28 and 29. Requirements & 28 and 29. Systems maintenance & 28 and 29. Testing // 33. Basic Software Applications & 33. Advanced Software Applications & 33. Requirements and System Selection // 30. information and communication technology / systems (applications) & 30. applied computer science & 30. system lifecycle management & 30. information and communication technology / systems (architecture) // 10. Operate equipment and software, trouble-shooting technical problems // 16. The platforms and software available according to clinical environment. // 23. Methods of practical informatics/computer science, especially on programming languages, software engineering, data structures, database management systems, information and system modelling tools, information systems theory and practice, knowledge engineering, (concept) representation and acquisition, software architectures // 24. Identifies and reports system process and functional issues (e.g. error messages, misdirections, device malfunctions, etc.) according to organizational policies and procedures. // |

*Article no. corresponds with Appendix 6 table.
Notes: Study which did not publish the full version of the framework (ie, Ayres *et al* [45]), competency categories which were aimed at non-health workers in a healthcare setting, competency categories which were irrelevant to digital competencies (e.g. ergonomics) were excluded from the analysis; Frameworks with ambiguous categories were excluded (e.g. computer skill, information literacy, information technology), unless pertaining category descriptions or competencies were provided; Frameworks with competency themes or statements encompassing several components of a competency, were carefully allocated to relevant domain(s) (e.g. competency statement ‘'The requirements for basic ICT, proficiency, quantitative analysis, and interpretation skills’ was allocated to competency domains ‘Analysis’ and ‘Informatics concepts and processes' respectively.
