# Supplementary figures and images for: A Digitally Competent Health Workforce: Scoping Review of Educational Frameworks
Source: J Med Internet Res. 2020 Nov 5;22(11):e22706. doi: 10.2196/22706 (PMC7677019; doi:10.2196/22706)

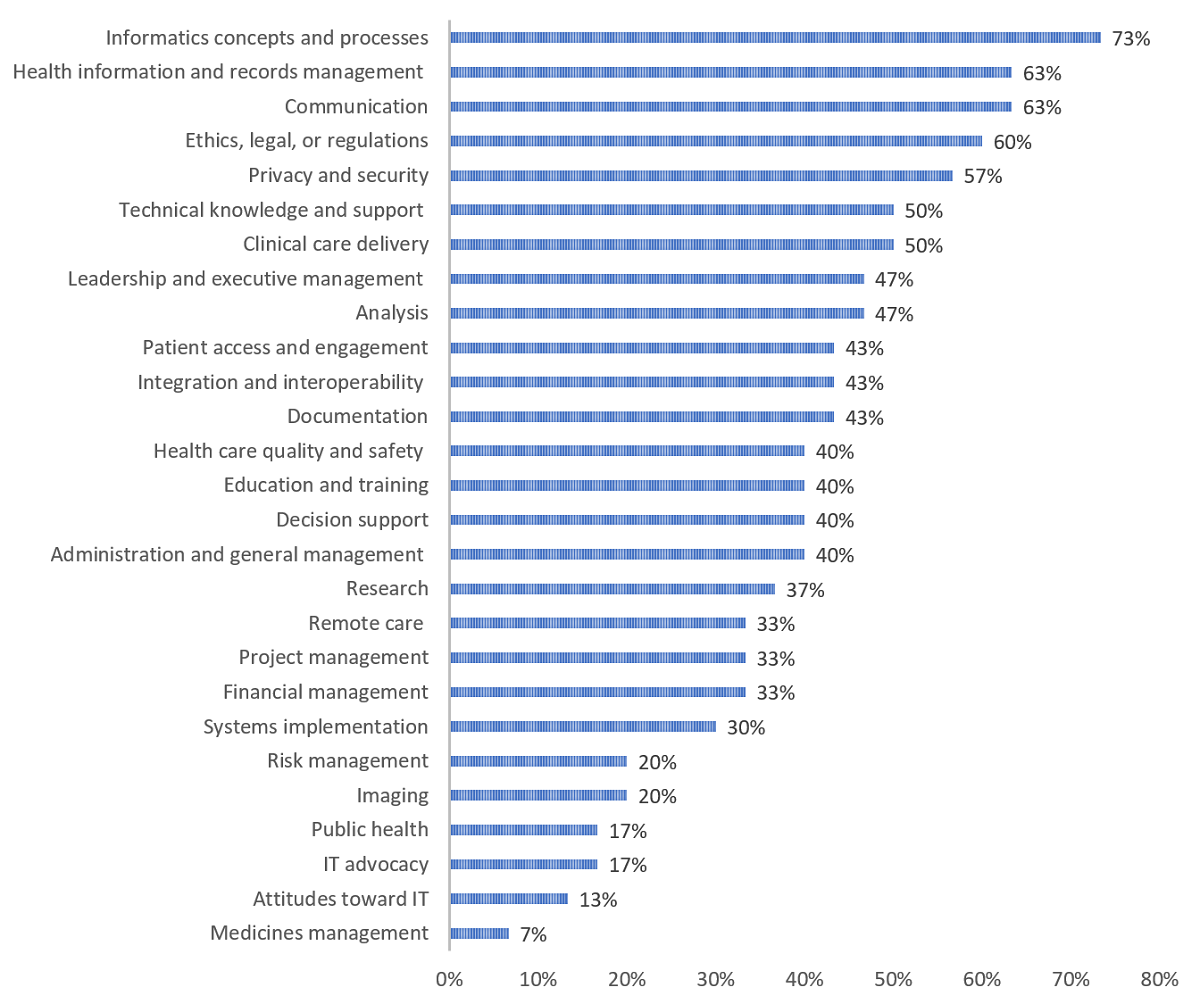

Supplement: Multimedia Appendix 8 [file jmir_v22i11e22706_app8.png]
